# Structural Origin of the Improved Ionic Conductance in CeO_2_–ZrO_2_–CeO_2_ Triple-Layer Electrolytes for Thin-Film Solid Oxide Fuel Cells

**DOI:** 10.1021/acsnano.6c02036

**Published:** 2026-07-21

**Authors:** Dongha Kim, Minyae Moon, Hangyeol Jung, Seongkook Oh, Cheol-Hwee Shim, Sungeun Yang, Jong-Ho Lee, Ji-Won Son, Deok-Hwang Kwon

**Affiliations:** † Center for Energy Materials Research, Clean Energy Research Division, Korea Institute of Science and Technology (KIST), Seoul 02792, Korea; ‡ Department of Energy Environment Policy and Technology, Graduate School of Energy and Environment (KU-KIST Green School), Korea University, Seoul 02792, Korea; § Division of Nano & Information Technology, KIST School, University of Science and Technology, Seoul 02792, Korea; ∥ Advanced Analysis and Data Center, Korea Institute of Science and Technology (KIST), Seoul 02792, Korea

**Keywords:** SOFC, electrolyte, columnar structure, ion conductor, low-temperature deposition

## Abstract

To lower the operation temperature of solid oxide fuel cells, thin-film electrolytes have attracted considerable attention, but their chemical instability and mechanical defects hinder their performance. To address these issues, gadolinia-doped ceria (GDC)–yttria-stabilized zirconia (YSZ)–GDC trilayer (CZC) thin-film electrolytes have been developed, wherein the fuel-side GDC layer provides high ionic conductivity and densifies the YSZ, and the cathode-side GDC chemically protects the YSZ electrolyte from the lanthanum strontium cobaltite cathode. Electrochemical impedance spectroscopy shows that the measured ionic conductance of CZC electrolytes is 3–4 times higher than the simple sum of the single-layer conductance of YSZ and GDC in series. This enhancement does not depend on the growth temperature, as low-temperature-deposited then annealed films perform comparably to their high-temperature-grown counterparts. X-ray diffraction and electron microscopy reveal that the YSZ layer in the CZC electrolytes forms highly continuous columnar grains with a markedly decreased grain boundary density, minimizing lattice discontinuities along the ion transport pathways. Grain-orientation mapping confirms predominantly low-angle columnar boundaries rather than the highly misoriented interfaces that would impede ion transport. These observations suggest that the CZC trilayer conductance is enhanced by the single-crystal-like columnar growth of YSZ, facilitated by its crystallographic compatibility with the underlying GDC. Collectively, these findings establish the practicality of CZC trilayers as a high-performance thin-film electrolyte operable at lower temperatures, providing microstructural design guidelines for interface-engineered solid oxide ion conductors. This approach highlights how interface-guided microstructural engineering can unlock unexpected transport properties in such conductors, offering broad implications for next-generation energy devices.

## Graphical abstract

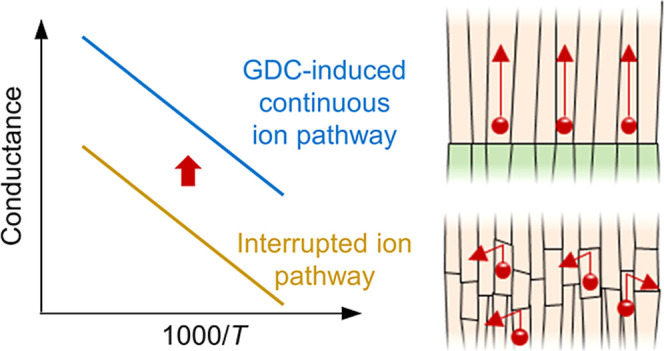

The escalating climate crisis has intensified the global demand for carbon-free energy technologies. Solid oxide cells, including solid oxide fuel cells (SOFCs) and solid oxide electrolysis cells (SOECs), are key technologies for sustainable energy conversion and storage. SOFCs directly convert chemical energy into electricity, whereas SOECs enable high-efficiency electrochemical conversion of steam and/or carbon dioxide into fuels or chemical feedstocks. Together, these technologies play a central role in achieving a sustainable, carbon-neutral energy cycle. In this context, the present study focuses on SOFCs, which stand out for their high energy conversion efficiency and exceptional fuel flexibility, making them suitable for a wide range of applications, from stationary power generation to auxiliary power units.^1^

Although SOFCs are considered a promising solution for sustainable energy conversion and storage, their high operating temperatures (typically 700–1000 °C) continue to limit their broader application.^2^ Such elevated temperatures require expensive heat-resistant materials and lead to several critical challenges, including prolonged thermal start-up and cool-down times, accelerated degradation mechanisms (e.g., nickel coarsening in the anode, chromium poisoning in the cathode, electrolyte densification, and interfacial delamination), and thermal stress during cycling.^3,4^ These issues collectively undermine the long-term reliability of SOFCs.

Lowering the operating temperature can mitigate these problems by enabling the use of cost-effective materials, improving durability, and suppressing side reactions.^2^ However, reducing the operating temperature may significantly degrade the cell performance owing to sluggish ion transport kinetics, particularly in oxide electrolytes. Thin-film SOFCs (TF-SOFCs) offer a promising pathway to overcome these challenges by employing nanometer-scale electrolyte layers, typically on the order of hundreds of nanometers, fabricated using physical vapor deposition (PVD) techniques such as sputtering or pulsed laser deposition (PLD). These thin layers effectively shorten the ionic conduction path and decrease the ohmic polarization, thereby enhancing the overall device performance, even at intermediate temperatures.^5–11^

PVD techniques not only reduce ohmic polarization but also enhance ionic conductivity by modulating grain boundary barriers more effectively than conventional bulk processes. In certain cases, they further enable epitaxial growth, which facilitates the fundamental investigation of ion transport mechanisms.^12,13^ By enabling the design of tailored nanoscale architectures, this nanostructural control has revealed the latent potential of metal oxide ion conductors. Consequently, thin-film-based optimization supports high electrochemical performance, even at lower operating temperatures.^14–16^

However, achieving high performance using thin electrolytes introduces challenges. To ensure gas-tightness and electronic insulation across a thin membrane, the electrically resistive thin layer must be fully dense and pinhole-free. Meeting these constraints has driven the research on electrolyte to evolve from single-layer yttria-stabilized zirconia (YSZ)^8,9,17^ to bilayer gadolinia-doped ceria (GDC)/YSZ^5,11,18,19^ and ultimately trilayer GDC/YSZ/GDC (CZC) electrolyte^20–23^ configurations. The CZC trilayer structure offers multiple functional advantages.^20^ The bottom GDC layer is exposed to the fuel electrode and has higher ionic conductivity, and it promotes the densification of the overlying YSZ while decreasing the overall resistance. The top GDC layer acts as a chemical barrier to prevent detrimental interfacial reactions between the YSZ electrolyte and the lanthanum strontium cobaltite cathode,^24–27^ which can otherwise form ion-insulating Sr–Zr–O secondary phases at high processing temperatures.^28,29^

The thin-film CZC electrolyte has primarily been valued for its chemical and mechanical stability thus far. However, we observed a surprising enhancement in the ionic conductance, exceeding the calculated values based on the intrinsic ionic conductivities of the individual YSZ and GDC components, regardless of the growth temperature. Previously, we found that a CZC cell containing an electrolyte grown at low temperature followed by postannealing exhibited an electrochemical performance comparable to that of a reference cell with a high-temperature-grown CZC electrolyte. Specifically, both cells showed similar electrochemical characteristics in terms of the peak power density. Such performance levels are typically unattainable via conventional bulk processing methods,^30^ suggesting that mechanisms beyond classical ohmic scaling contributed to the improved performance. Although the present work focuses on TF-SOFC operation, reducing electrolyte ohmic resistance is also highly relevant to SOEC operation, where ionic transport losses become increasingly critical under high-current-density electrolysis conditions. Therefore, the CZC trilayer design, which promotes continuous columnar ion-transport pathways and reduced grain-boundary-related transport limitations, may also provide a useful structural strategy for thin-film SOEC electrolytes.

To elucidate the origins of this performance enhancement, we systematically compared six electrolyte configurations, each characterized by distinct grain boundary densities and underlying layer compositions achieved through controlled deposition and postannealing conditions. These configurations are hereafter denoted by YSZ–300+700, YSZ–700, GDC–300+700, GDC–700, CZC–300+700, and CZC–700, where “300+700” refers to samples grown at 300 °C followed by a postannealing treatment at 700 °C, whereas “700” indicates samples grown directly at the high temperature of 700 °C without postannealing.

We characterized the films through X-ray diffraction (XRD), scanning electron microscopy (SEM), and transmission electron microscopy (TEM), including ASTAR (also referred to as automated crystal orientation mapping in TEM (ACOM-TEM)^31^) imaging to visualize the crystallographic orientation across an adequate field of view. These analyses revealed that the YSZ grown on GDC forms highly continuous out-of-plane columns with suppressed internal grain-boundary interruptions. High-resolution TEM (HR-TEM) further confirmed the epitaxial relationship between the YSZ and the underlying GDC.

Based on these observations, the enhanced ion conduction in CZC is attributed to GDC-assisted epitaxial templating of YSZ, which forms continuous out-of-plane ion-transport pathways with minimal internal grain-boundary interruption. This decreased the grain boundary density and formed relatively coherent columnar boundaries. Because ions are transported in TF-SOFCs in the out-of-plane direction, the columnar boundaries do not significantly hinder conduction because they are aligned along these transport pathways. Thus, optimizing the microstructure of thin-film electrolytes through epitaxial growth and layer engineering provides a powerful route for improving the SOFC performance at lower temperatures. Through this study, we clarified the structural origins of the enhanced conductance of CZC, offering insights for designing next-generation TF-SOFC electrolytes with improved performance and durability.

## Results and Discussion

### Enhanced Ionic Conductance in CZC

To investigate the origin of the unexpectedly high conductance of the CZC relative to the calculated values, we prepared a set of samples designed to decouple the effects of the deposition temperature and underlying layers, which are the most critical factors influencing thin film growth. The deposition temperature directly alters the relaxation time of the clusters adsorbed on the substrate surface, thereby affecting the initial nucleation process and growth features of the film. We employed two different deposition processes, single-step, high-temperature growth at 700 °C and low-temperature growth at 300 °C followed by annealing at 700 °C, based on a prior study wherein films prepared under these conditions exhibited comparable electrochemical performance. Because the underlying layer can provide a crystal field that influences the crystallographic orientation of the film, single-layer YSZ and GDC samples were also prepared to isolate this effect.

All films were deposited on Nb-doped SrTiO_3_ (Nb:STO) substrates using PLD.^30^ For the out-of-plane conductance measurements, electrically conductive Nb:STO served as the bottom electrode, while a top Pt electrode with a diameter of 1 mm was sputtered onto the electrolyte films. This configuration avoided the false evaluations of the conductance that may occur in in-plane measurements.^32–34^ Electrochemical impedance spectroscopy (EIS) was conducted across a range of temperatures, allowing the construction of Arrhenius plots for each electrolyte type (Figure 1a).^35,36^ EIS results were fitted with an equivalent circuit model composed of a parallel RC element for the electrolyte, a series resistance of the lead wires, and a Warburg element associated with the dense top Pt electrode (Figures 1b and S2). Because of the multilayer structure of CZC, we compared their total ionic conductance rather than their conductivity, as the overall resistance is dominated by the YSZ layer within the limited film thickness, making conductance a more appropriate metric than the intrinsic property of conductivity. The total resistance was computed as *R*_CZC_ = *R*_GDC_(150 nm) + *R*_YSZ_(400 nm) + *R*_GDC_(700 nm) using the relationship $G=\frac{1}{R}$ , where *G* is the conductance. The resistances of the individual GDC and YSZ layers were directly measured using the corresponding single-layer samples. The calculated values are labeled “CZC^cal^” in Figure 1, while the experimentally measured conductance of CZC is denoted as “CZC^exp^.”

**Figure 1:**
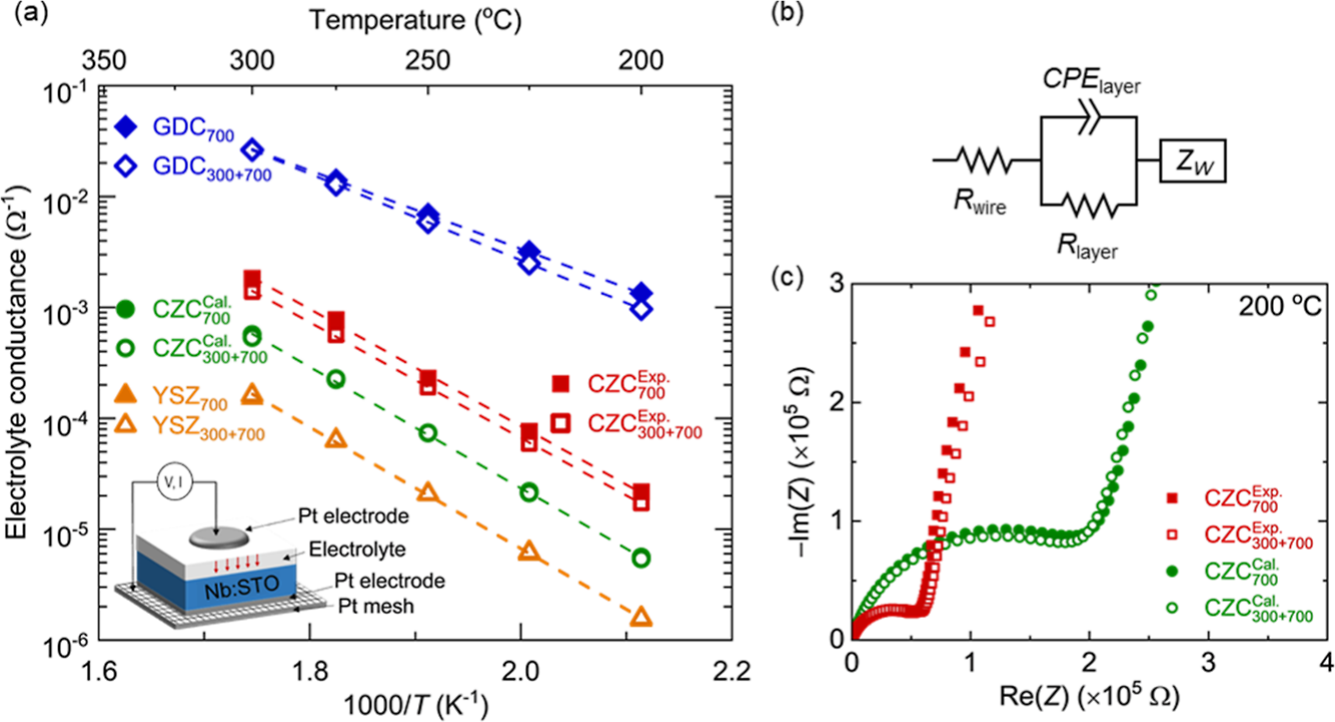
Temperature dependence of the ion conductance of 1.25 μm-thick electrolytes. (a) Arrhenius plots comparing the ionic conductances of 1.25 μm-thick GDC (blue diamonds), YSZ (orange triangles) single-layer, and CZC trilayer electrolytes deposited at high (closed symbols) and low (open symbols) temperatures. The CZC configuration consists of a 150 nm GDC/400 nm YSZ/700 nm GDC trilayer. “CZC^exp^” (red squares) denotes the experimentally measured conductance, while “CZC^cal^” (green circles) represents the calculated conductance based on the individual layer thicknesses and conductivities (see Tables S1 and S2, and Figure S1a). Inset: two-probe configuration used for electrochemical impedance spectroscopy (EIS). (b) Equivalent electrical circuit model used for EIS analysis. (c) Nyquist plots of the CZC^exp^ and CZC^cal^ at 200 °C.

It should be noted that thin-film electrolytes exhibit fundamentally different behavior in EIS spectra compared to conventional bulk ceramics, in which the semicircular arcs associated with bulk and grain boundary responses are typically well separated. In the present out-of-plane thin-film geometry, these responses may partially overlap because the characteristic relaxation frequencies of the bulk and grain-boundary components become insufficiently separated. In general, bulk responses in Nyquist plots exhibit capacitances on the order of ∼10^–12^ F, whereas grain boundary responses typically observed in the ∼10^–11^ to 10^–8^ F range. However, compared with conventional ceramic electrolytes with transport lengths of several to hundreds of micrometers or more, the present out-of-plane thin-film geometry contains only a submicrometer-thick YSZ transport layer (∼400 nm). This greatly reduces the number of serial grain-boundary interruptions along the transport direction and can substantially decrease the grain-boundary resistance contribution. Therefore, even if the grain-boundary capacitance remains larger than the bulk capacitance, the corresponding relaxation time constant (*τ* = *RC*) and characteristic frequency can approach those of the bulk response. Consequently, the bulk and grain-boundary contributions can become strongly overlapped in the impedance spectra, particularly when the high-frequency arcs are nonideal or deformed. Therefore, the electrochemical performance of the samples was evaluated primarily based on the total resistance/conductance, rather than attempting rigorous quantitative separation of individual bulk and grain-boundary contributions.

Based on total resistance, we compared the various configurations of electrolytes grown at different temperatures and configurations. Remarkably, both CZC films (red squares) exhibited ionic conductance values that were approximately three to four times higher than the calculated values based on the individual GDC and YSZ layers (CZC^cal^, green circles), as shown in Figure 1a,c (detail values in Tables S1 and S2). This unexpected enhancement suggests that the performance of the trilayer CZC cannot be explained as simply the sum of the behavior of the individual components. For the YSZ films, both YSZ–700 and YSZ–300+700 (orange triangles) exhibited nearly identical ionic conductance and activation energies of ∼1.13 eV, indicating that the growth temperature had minimal impact. In contrast, the GDC films (blue diamonds) showed a more pronounced difference: GDC–700 had an activation energy of 0.73 eV, whereas that of GDC–300+700 increased to 0.81 eV, accompanied by a slight drop in conductance, which was approximately 2-fold lower at 200 °C. Nevertheless, the ionic conductivities of the YSZ and GDC films were comparable to previously reported values (Figure S1).^37,38^ The higher ionic conductance of GDC–700 suggests that GDC was more sensitive to the growth temperature and annealing history than YSZ and that high-temperature deposition was favorable for achieving high ionic conductivity in the GDC electrolyte.

Notably, both CZC–700 (red closed squares) and CZC–300+700 (red open squares) exhibited similar ionic conductance and activation energies (1.06 and 1.10 eV, respectively). Although the ionic conductance of GDC–300+700 was lower than that of GDC–700, the total conductance of the CZC–300+700 structure remains comparable to its high-temperature-deposited counterpart. This is primarily because the YSZ layer, despite being only ∼400 nm thick (approximately half the thickness of the combined GDC layers), had an ionic conductivity that was orders of magnitude lower. As a result, contributed nearly 99% of the total resistance, thus dominating this feature. In other words, the YSZ layer constituted the primary bottleneck for ionic conduction within the CZC structure. Thus, our subsequent investigation focused on the structural differences in the YSZ layers between different configurations.

To investigate the underlying factors contributing to the distinctive ionic transport behavior of the electrolytes—especially the unexpectedly high conductance observed in the CZC structure relative to the theoretical predictions—we analyzed their microstructures in detail using XRD, SEM, and TEM. In particular, ASTAR orientation mapping was employed to elucidate the grain orientations and epitaxial relationships within the layered structure, which play critical roles in enhancing ionic transport.

### Crystal Structure and Cross-Sectional Morphology

First, XRD and cross-sectional SEM were used to assess the overall crystallographic texture and growth morphology of the GDC, YSZ, and CZC films (Figure 2). The *θ*–2*θ* XRD patterns shows that the out-of-plane orientation of each layer was strongly influenced by both the growth temperature and the crystalline texture of the underlying layer. Films deposited at 700 °C exhibit sharp diffraction peaks dominated by the (200) reflection, whereas those deposited at 300 °C and postannealed at 700 °C show a weaker texture and additional reflections, indicating a more randomized crystallographic orientation.

**Figure 2:**
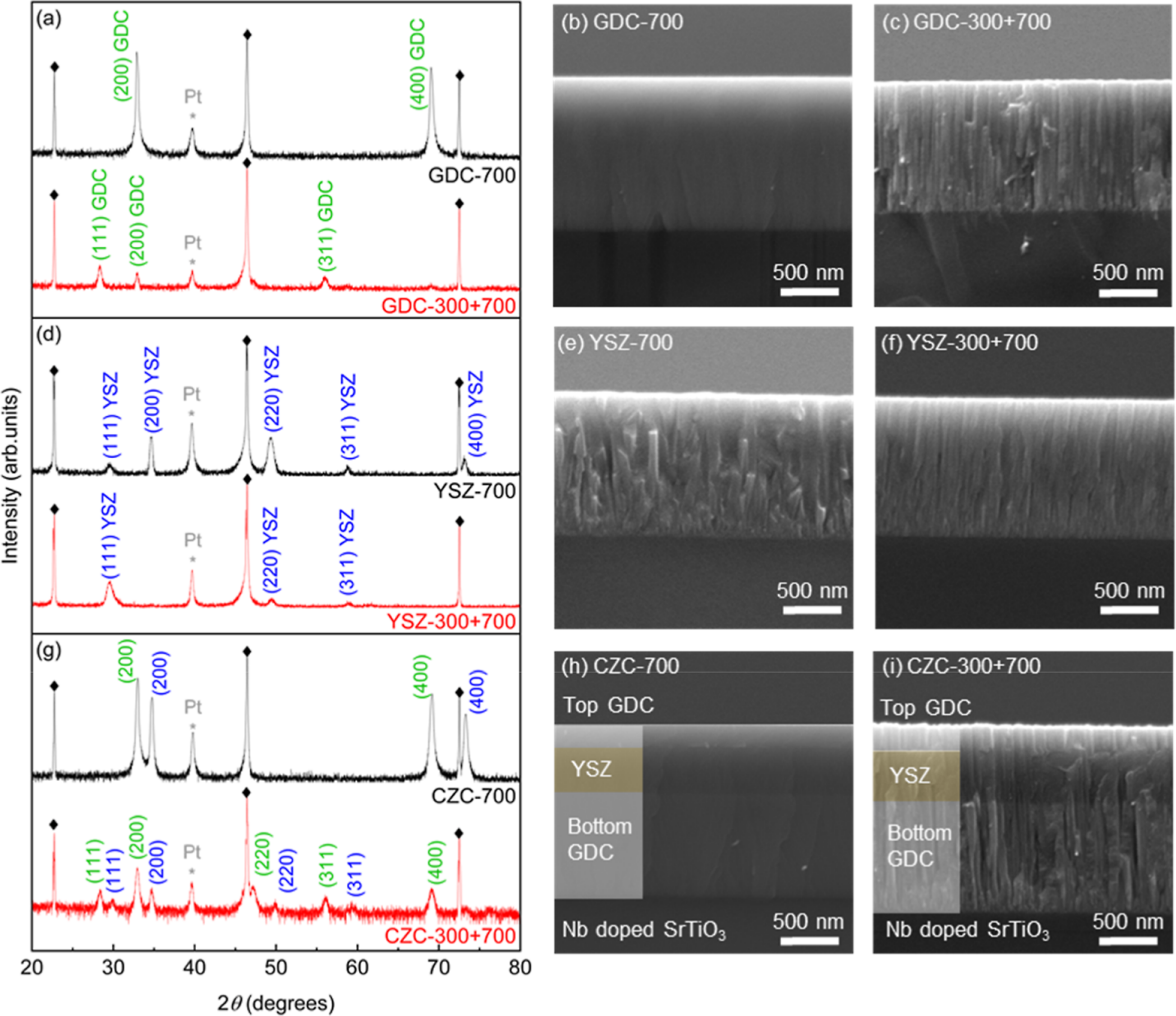
XRD patterns and SEM images of GDC, YSZ, and GDC/YSZ/GDC (CZC) layers grown on Nb-doped SrTiO_3_ (Nb:STO) substrate. (a–c) XRD and SEM of GDC layers grown on Nb:STO substrate at 700 °C (GDC–700) and at 300 °C with postannealing at 700 °C (GDC–300+700). Corresponding characterization of (d–f) YSZ and (g–i) trilayer CZC layers grown in the same manner. Common peaks in each XRD pattern indicate the (00*l*) family of planes in Nb:STO (black diamonds) and the (111) Pt plane from the top electrode (gray stars). The GDC and YSZ patterns were indexed to PDF 04-002-6160 and 04-015-2373, respectively.^39^

This temperature-dependent behavior reflects a transition from substrate-driven epitaxial growth with high adatom mobility to surface-energy-dominated growth at lower temperatures. At elevated temperatures, both GDC and YSZ preferentially adopt the (200) orientation imposed by the (001)-oriented Nb:STO substrate. Cubic fluorite GDC and YSZ have lattice parameters of 5.39 and 5.14 Å, respectively, whereas that of the Nb:STO substrate is 3.905 Å. Epitaxial alignment was enabled by a 45° in-plane rotation (Figure S3), satisfying the geometric relation $3.905\times \sqrt{2}\approx 5.222\;Å$ . In contrast, decreased surface diffusion at lower deposition temperature limited the crystallographic alignment, leading to the emergence of alternative orientations such as (111), a trend consistently observed in single-layer GDC and YSZ films.

Notably, the crystallographic response of YSZ changed markedly when incorporated into the CZC trilayer structure. In both CZC–700 and CZC–300+700, the YSZ layer retained a pronounced (200) reflection, with an intensity exceeding that of the corresponding single-layer YSZ films deposited under identical conditions. This persistence of the (200) texture, even after low-temperature growth followed by postannealing, indicates that the YSZ orientation was stabilized by the adjacent GDC layers rather than solely by thermal effects. This effect was not observed in single-layer YSZ, highlighting the critical role of the underlying GDC layer. In particular, the bottom GDC layer served as a crystallographic template, which decreased the effective lattice mismatch to ∼4.64%, in contrast with direct growth on Nb:STO (∼6.88%), thereby promoting coherent (200)-oriented growth (Figure S3). This behavior is consistent with a cube-on-cube epitaxial relationship between the two fluorite structures.

Cross-sectional SEM images provide complementary morphological insights. Although single-layer YSZ and GDC films exhibited temperature-dependent columnar growth, the YSZ layer within CZC adopted a smoother, more uniform columnar morphology closely resembling that of GDC. Notably, the rough, V-shaped columnar features characteristic of single-layer YSZ–700 was noticeably suppressed in CZC–700, consistent with the templated growth inferred from XRD.

As YSZ and GDC possess cubic fluorite structures that are inherently isotropic, their crystallographic orientation alone has a negligible direct impact on ionic conductance. Instead, ionic transport is governed primarily by microstructural factors such as grain size, crystallinity, and the density and distribution of grain boundaries. These microstructural features are especially critical in determining the total ionic conductance, as grain boundaries act as resistive barriers along the ion transport pathways between the electrodes and electrolytes. The XRD and SEM results therefore prompted a more detailed investigation of the local microstructure using TEM and ASTAR, as presented in the following section.

### Grain Analysis of the YSZ Layer

Because the YSZ layer constitutes the dominant bottleneck for ionic transport in the CZC architecture, we first focused on how the grain boundary population and spatial distribution evolved under different growth configurations. The central question was whether the multilayer CZC structure modified the microstructural pathway for ion migration relative to that in single-layer YSZ films. To address this, TEM combined with ASTAR orientation mapping was employed to visualize the grain structure and crystallographic continuity across the film thickness.

Single-layer YSZ films provided a baseline for grain-boundary-controlled transport. The TEM image and ASTAR mapping of YSZ–300+700 (Figure 3a,b, respectively) reveal very narrow columnar grains (∼20 nm wide), within which individual grains were only ∼200 nm tall and became progressively finer toward the substrate interface. This indicates limited grain coalescence during early growth, leading to a dense population of nanometer-scale grains near the interface. In YSZ–700 (Figure 3c,d), higher growth temperature promoted grain coalescence within the first ∼100 nm of the film, resulting in wider, V-shaped columns typical of high-temperature columnar growth. Despite these differences in upper-layer morphology, however, both films shared a critical structural feature near the substrate.

**Figure 3:**
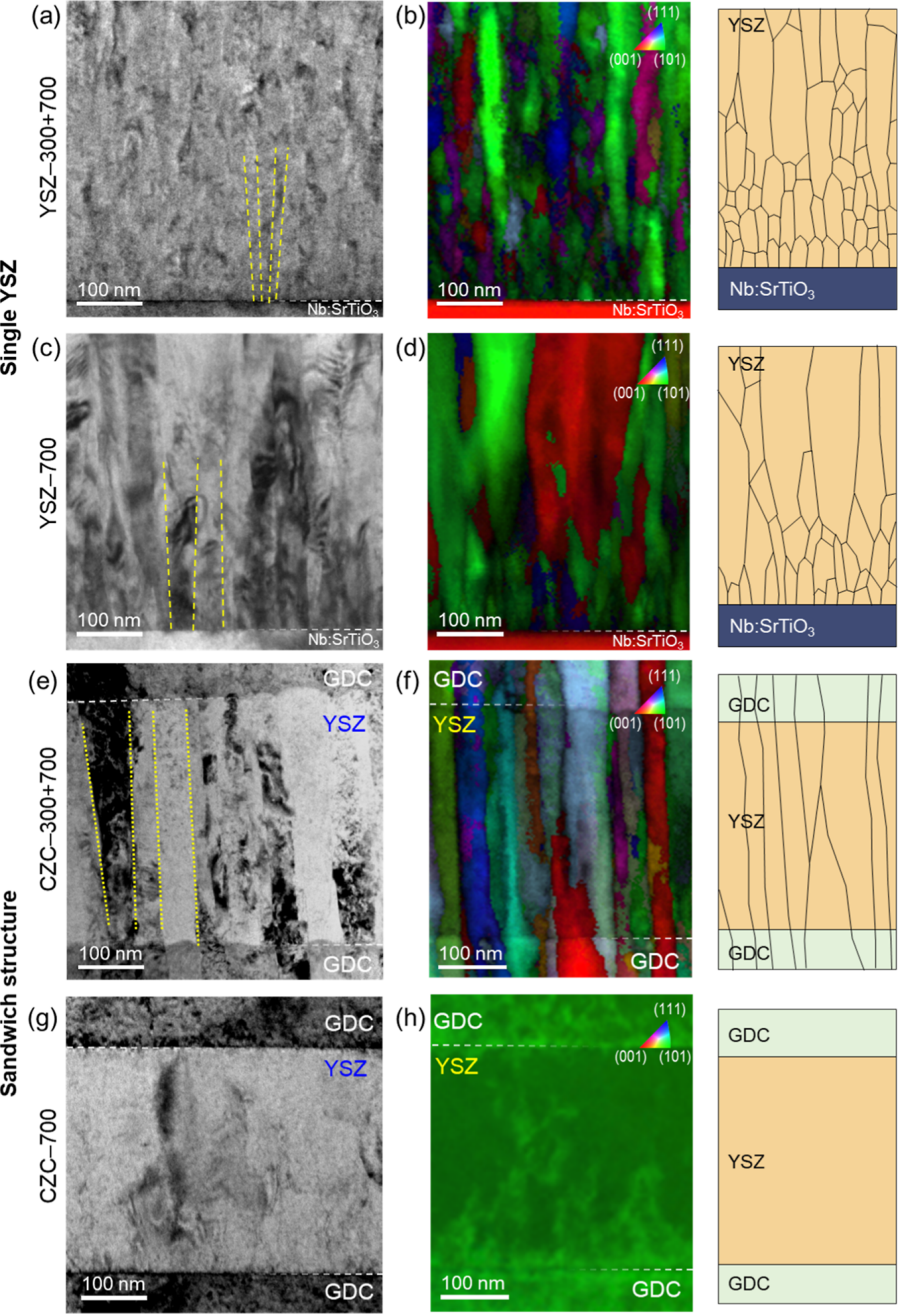
TEM images, ASTAR crystal orientation maps, and schematics of the YSZ layer in different samples. (a,b) YSZ–300 + 700, (c,d) YSZ–700, (e,f) CZC–300+700, and (g,h) CZC–700. The thin-film electrolytes were grown on Nb:STO. Insets (b,d,f,h): color codes indicating the crystallographic grain orientation along the beam direction, aligned with the (001) orientation of Nb:STO.

Specifically, a fast Fourier transform (FFT) analysis of enlarged TEM images (Figure S4) indicated that both YSZ–700 and YSZ–300+700 developed a dense population of nanometer-sized grains within the bottom ∼200–300 nm of the film, as evidenced by ring-like diffraction patterns. Because oxygen ions migrated along the out-of-plane direction, these interfacial nanograins introduced a high density of serial grain boundaries that dominated the total ionic resistance, rendering the variations in the upper-layer morphology largely inconsequential. This explains the similar ionic conductance and activation energies observed for the two films, despite their distinct columnar structures (Figure 1).

By comparison, single-layer GDC films exhibited substantially fewer grain boundaries. GDC–700 was nearly single-crystalline, as evidenced by the sharp XRD peaks (Figure 2a) and uniform ASTAR orientation map (Figure S5a,b), whereas GDC–300+700 formed wider columnar grains (∼20–50 nm) than the YSZ films because of favorable lattice matching with the Nb:STO substrate. Despite its larger columns, GDC–300+700 contained more grain boundaries than GDC–700 and therefore exhibited lower ionic conductance and a higher activation energy (Figure S1).^40^

The most striking structural transformation occurred in the sandwiched YSZ layer within the CZC trilayer. In CZC–300+700, the YSZ layer grew epitaxially on the underlying GDC, forming vertically aligned columns that extended coherently from the bottom GDC layer through the YSZ to the top GDC layer,^41,42^ as highlighted by the yellow dashed guides in Figure 3e. The ASTAR orientation map (Figure 3f) further reveals minimal internal grain segmentation in the YSZ layer and highly continuous crystallographic orientation along the out-of-plane transport direction. In many columns, identically colored domains extend continuously across all three layers, demonstrating that the crystallographic orientation was preserved throughout the trilayer stack (Figure 3f). This behavior contrasts sharply with that of the single-layer YSZ–300+700 film, wherein frequent color discontinuities near the interface indicate randomly oriented nanograins. Minor variations in ASTAR coloration are attributed to grain overlap along the beam direction during TEM imaging.

In CZC–700, the YSZ layer contained columnar grains with near single-crystalline continuity, as observed in low-magnification TEM images (Figure 3g). Corresponding selected-area diffraction patterns (Figure S6) show overlapping spots attributed to YSZ and the adjacent GDC, with minor *d*-spacing variations due to lattice mismatch, suggesting coherent epitaxial growth without grain disruption. Consistently, ASTAR orientation map (Figure 3h) reveals uniform green domains spanning the bottom GDC, YSZ, and top GDC layers, confirming a shared crystallographic orientation with no evidence of intragranular misalignment or fine-grained segmentation.

In summary, the CZC configuration modifies the microstructural evolution of YSZ through GDC-assisted epitaxial templating, beyond what can be achieved by controlling the deposition temperature alone. Compared with single-layer YSZ films, which exhibit nanograin-rich interfacial regions and frequent crystallographic interruptions, the YSZ layer in the CZC trilayer maintains continuous columnar growth with suppressed interfacial renucleation and reduced grain-boundary interruption along the out-of-plane ion-transport direction. This behavior arose from the structural compatibility and modest lattice mismatch between YSZ and GDC, which facilitated epitaxial templating. The resulting decrease in the effective grain boundary density along the transport direction accounts for the enhanced ionic conductance of CZC electrolytes and their reduced sensitivity to the growth temperature, highlighting the role of GDC-assisted interfacial microstructural control in enhancing the ionic transport.

### Quantitative Comparison of YSZ in the CZC Structure

To further correlate the microstructure with the enhanced conductance, we performed a quantitative analysis of the YSZ columnar structure based on ASTAR/TEM observations (Figure 4). By statistically measuring the column width and uninterrupted column length for all samples, we directly compared the extent of grain-boundary interruption and crystallographic continuity along the out-of-plane ion-transport direction.

**Figure 4:**
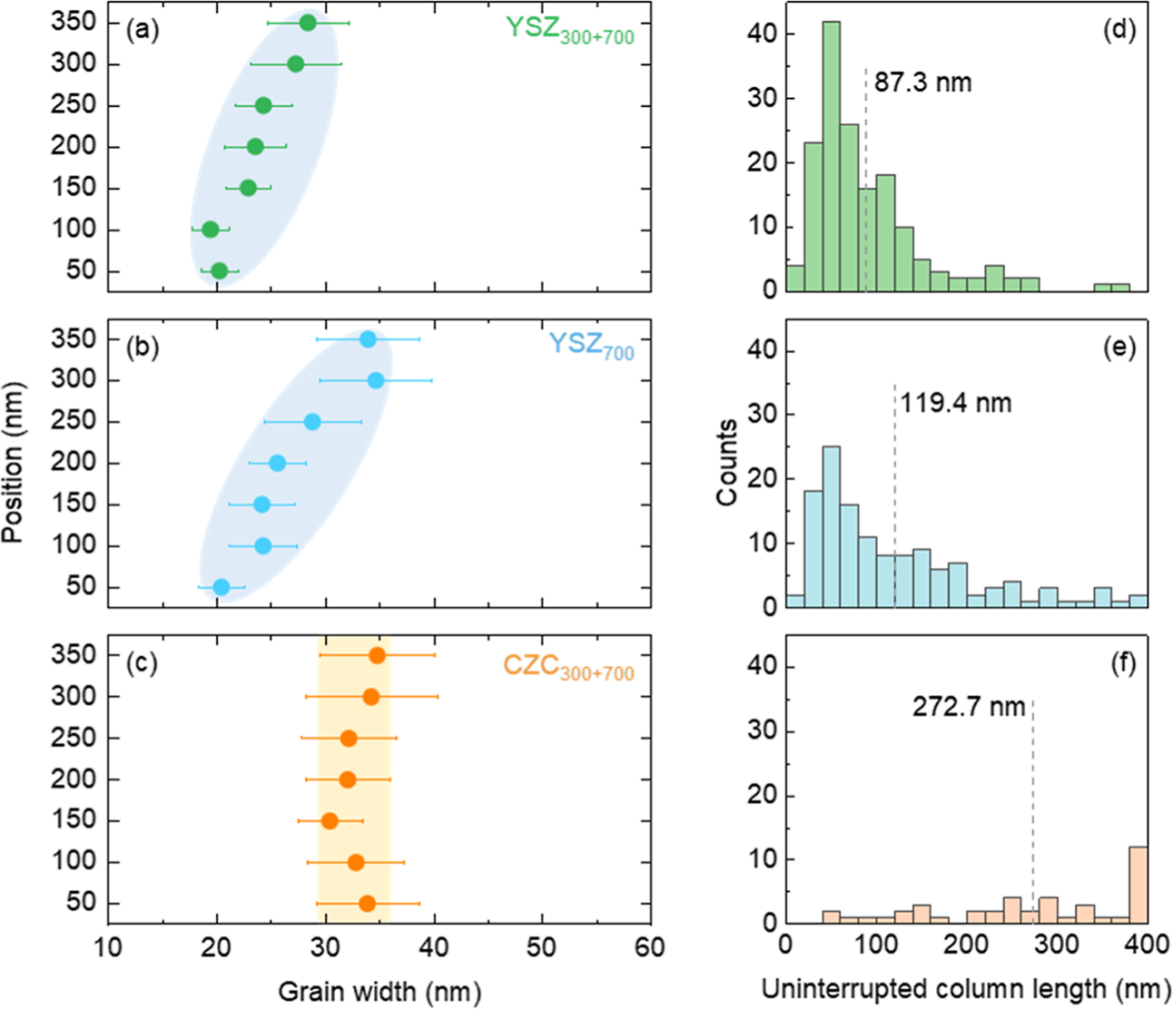
Quantitative comparison of the YSZ columnar microstructure based on ASTAR/TEM analysis. (a–c) Average lateral column width measured at different distances from the bottom interface for YSZ–300+700, YSZ–700, and CZC–300+700. The single-layer YSZ films exhibit relatively narrow columns near the interface that gradually widen with increasing film thickness, whereas CZC–300+700 maintains larger and more uniform column widths throughout the film thickness, indicating more continuous columnar growth. Error bars represent the standard deviation of the measured column widths. (d–f) Distributions of uninterrupted column length measured along the out-of-plane transport direction across the 400 nm-thick YSZ region for YSZ–300+700, YSZ–700, and CZC–300+700. The dashed lines indicate the average uninterrupted column length for each sample.

First, the lateral column width was evaluated as a function of distance from the interface within the 50–350 nm region (Figure 4a–c). In the single-layer YSZ films (Figure 4a,b), the columns were approximately 20 nm wide near the interface and gradually widened with increasing film thickness. In contrast, CZC–300+700 (Figure 4c) maintained relatively uniform column widths of ∼30–35 nm throughout the film thickness, indicating more continuous columnar growth. Notably, even between the two single-layer YSZ samples, YSZ–700 exhibited more rapid column coarsening than YSZ–300+700, confirming the strong influence of deposition temperature on grain growth kinetics. However, when YSZ was deposited on the underlying GDC layer, large column widths were established from the early growth stage even under the low-temperature deposition condition. These observations indicate that, although deposition temperature strongly influences the microstructure, the adjacent GDC layers in the CZC structure additionally suppress the formation of fine-grained interfacial regions.

Second, to evaluate the continuity of the out-of-plane transport pathways, distributions of uninterrupted column length were estimated across the 400 nm-thick YSZ region (Figure 4d–f). The average uninterrupted column lengths were approximately 87.3 nm for YSZ–300+700, 119.4 nm for YSZ–700, and 272.7 nm for CZC–300+700. In the single-layer YSZ films, short uninterrupted segments below 100 nm accounted for ∼80% of the measured paths in YSZ–300+700 and ∼60% in YSZ–700, indicating frequent grain-boundary interruptions along the out-of-plane direction. In contrast, such short segments accounted for only ∼10% in CZC–300+700, while ∼27% of the measured paths extended continuously across the full 400 nm-thick YSZ layer without observable interruption. These results indicate substantially reduced grain-boundary interruption along the out-of-plane transport direction in the CZC structure.

These quantitative microstructural differences provide a direct structural basis for the enhanced conductance of the CZC trilayer. In YSZ, grain-boundary transport resistance is generally much larger than bulk transport resistance; therefore, frequent grain-boundary interruptions within the 400 nm-thick YSZ transport layer can significantly increase the effective out-of-plane resistance. The single-layer YSZ films contain nanograin-rich interfacial regions and frequent grain-boundary interruptions, whereas many YSZ transport pathways in the CZC structure exhibit highly continuous columnar growth with few observable interruptions across the layer. This structural contrast explains why the experimentally measured CZC conductance exceeds the value calculated from the single-layer components: the CZC trilayer reduces the effective grain-boundary limitation within the YSZ layer by promoting continuous out-of-plane columnar pathways. This interpretation is further supported by comparison with reported single-crystal YSZ values, where the conductance of the CZC trilayer approaches the upper range of single-crystal YSZ (Figure S7).

Although the YSZ layer in CZC is not an ideal single-crystalline film, many YSZ columns exhibit nearly single-crystal-like crystallographic continuity along the out-of-plane direction. In this sense, the CZC structure can be described as quasi-single-crystalline at the column level, characterized by highly continuous columnar growth and suppressed internal grain-boundary interruption compared with the fragmented single-layer YSZ films.

### HR-TEM Analysis of YSZ in the CZC Structure

To further validate the crystallographic continuity inferred from the ASTAR orientation maps, HR-TEM analysis was performed on the CZC structures. The epitaxial relationship between GDC and YSZ previously observed in the CZC–700 sample was consistently observed across the entire sample (Figure S8a).

Because CZC–300+700 exhibited columnar grains, a more localized crystallographic analysis was conducted along individual columns at higher magnification (Figure S8b). Figure 5a focuses on the lower GDC/YSZ interface, which played a critical role in governing the subsequent growth of the YSZ layer. The TEM image reveals continuous columnar grains extending across the interface, as highlighted by dashed guides. FFT patterns acquired from the bottom GDC, the GDC/YSZ interface, and the YSZ layer (regions i–iii) exhibit identical diffraction spot arrangements, indicating a shared crystallographic orientation across these regions.

**Figure 5:**
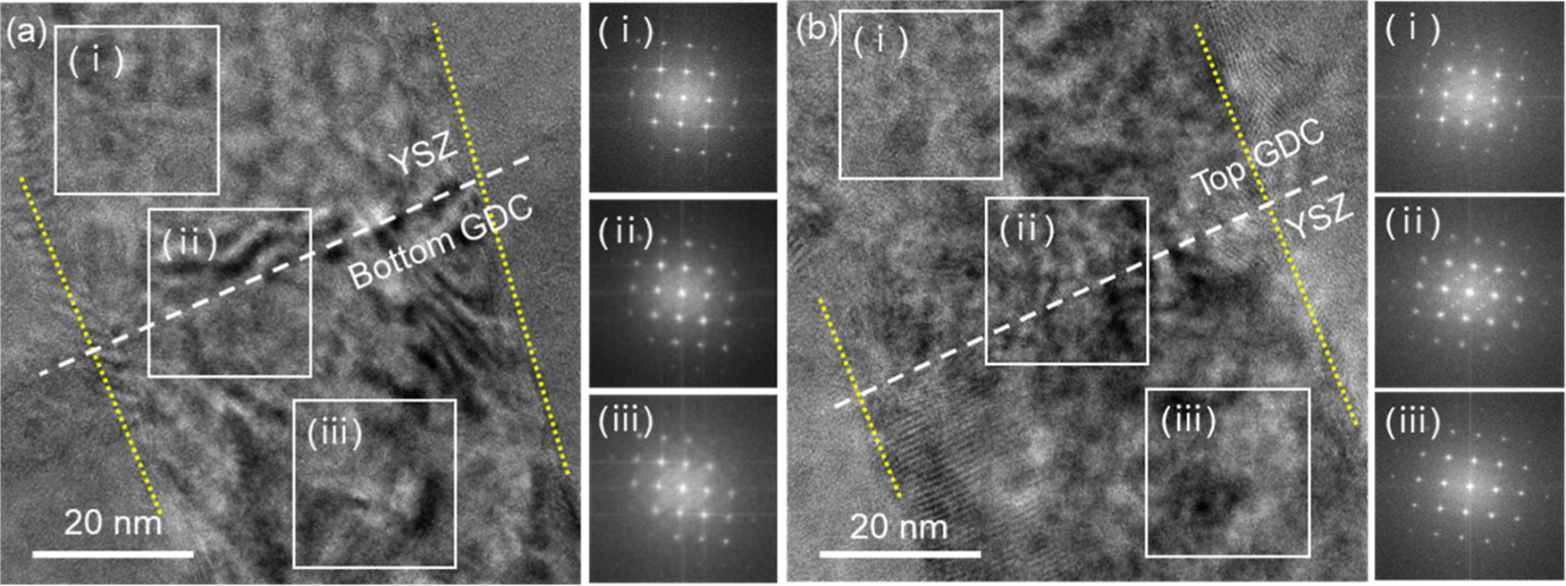
High-magnification TEM image and the corresponding FFT pattern of the GDC/YSZ interface in the CZC–300+700 sample. (a) Lower YSZ/GDC region and (b) upper GDC/YSZ interface, with FFT patterns obtained along the columnar direction.

A similar YSZ–GDC relationship was also observed at the upper GDC/YSZ interface (Figure 5b). The FFT patterns collected along the columnar direction from the YSZ layer, the GDC/YSZ interface, and the top GDC layer are identical to those obtained at the lower interface (Figure 5a), demonstrating that the YSZ layer maintained crystallographic continuity throughout the trilayer stack. These high-resolution results provided direct crystallographic evidence supporting the ASTAR orientation maps shown in Figure 3, which visualize the orientation continuity on a larger spatial scale.

Although the CZC–300+700 sample appeared macroscopically polycrystalline in the XRD patterns, the combined ASTAR and HR-TEM analyses revealed that at the columnar level, the YSZ layer formed a highly textured, epitaxially guided structure with minimal grain boundary disruption along the out-of-plane direction.

### Interpretation of the Enhanced Ionic Conductance Based on the Grain Orientation

We systematically evaluated the ionic conductance of thin-film electrolytes—YSZ, GDC, and CZC trilayers—using EIS. Notably, the ionic conductances of both CZC–700 and CZC–300+700 were several times higher than that of single-layer YSZ, regardless of the growth temperature. This enhancement prompted us to investigate the microstructural origins of the observed performance differences, with a particular focus on the YSZ layer, which dominated the total resistance of the trilayer owing to its intrinsically low ionic conductivity (Figure S1).^37,38^

Structural analyses indicated that both GDC and YSZ typically exhibited columnar morphologies, as evidenced by the SEM and TEM images. The column width increased at the higher growth temperature, attributable to higher mobility of adatoms and grain coalescence. Following high temperature deposition, the GDC layer on the Nb:STO substrates was nearly single crystalline, likely due to its minimal lattice mismatch and favorable epitaxial relationship with the substrate. A similar trend was observed for the CZC electrolyte layer in the tested full cell (Figure S9), indicating that the key microstructural feature identified in this study—namely, coherent columnar growth along the ion-transport direction—can also be realized in a practical cell geometry. Importantly, the corresponding anode-supported CZC full cell exhibited low ohmic ASR values of 0.072 Ω·cm^2^ at 500 °C and 0.018 Ω·cm^2^ at 650 °C, demonstrating that the modified CZC microstructure can maintain low ohmic loss under SOFC-relevant operating conditions.^30^ Notably, the estimated ASR at 600 °C is ∼ 0.027 Ω·cm^2^, lower than the reported 0.06 Ω·cm^2^ for a CZ bilayer electrolyte cell, despite the thicker YSZ layer in the CZC structure.^43^

Because ionic conduction predominantly proceeds in the out-of-plane direction, the nature and distribution of grain boundaries along this pathway critically determine the overall conductance. Vertical columnar boundaries aligned with the transport direction impose minimal resistance, whereas horizontal or strongly misoriented boundaries disrupt lattice continuity and act as effective barriers. In this context, the YSZ layer embedded within the CZC structure exhibits a fundamentally different microstructure compared with single-layer YSZ films. The ASTAR orientation maps and HR-TEM images show YSZ grains grown on GDC exhibit highly continuous crystallographic orientation along the transport direction, with suppressed internal grain-boundary interruptions. In contrast, the single-layer YSZ grown without the GDC layer contained multiple grain segments and fragmented columns, resulting in a high density of interfacial and intragrain boundaries. The improved crystallinity in CZC is attributed to the favorable lattice matching and structural isomorphism between YSZ and GDC, both of which adopted cubic fluorite structures with relatively low lattice mismatch, enabling epitaxial alignment and vertically continuous, low-defect conduction pathways. Since the electrolyte layers investigated here are several hundred nanometers thick, substantially exceeding the typical strain-retention thickness reported for oxide thin films, which is generally limited to several to several tens of nanometers, the observed conductance enhancement is more likely associated with differences in microstructure and grain-boundary characteristics rather than epitaxial strain effects.

Although lattice-mismatch-related defects may, in principle, exist locally at the GDC/YSZ heterointerfaces, TEM/ASTAR analyses did not reveal severe crystallographic disruption, near-interface renucleation, or loss of columnar continuity. Instead, YSZ maintained continuous columnar growth templated by the adjacent GDC layers. Combined with the conductance enhancement beyond the calculated series value, these observations indicate that additional GDC/YSZ interfacial resistance is not the dominant factor governing ionic transport in the present CZC electrolyte.

Taken together, these results indicate that the enhanced conductance of the CZC trilayer primarily originates from the GDC-assisted reduction of grain-boundary limitation within the YSZ layer, enabling more continuous out-of-plane transport pathways that approach the upper range of reported single-crystalline YSZ conductance.

Arrhenius plots (Figure 1) show that the activation energies of the YSZ and CZC layers remained similar across growth temperatures (300 and 700 °C), consistent with the marginal difference in the activation energy between bulk and grain boundary conduction in YSZ.^13,33,44,45^ Thus, the conductance enhancement observed in CZC was not due to a decrease in the activation energy but rather to an increase in the pre-exponential factor (σ_0_) in the Arrhenius relation

$$\sigma =\sigma_{0}\exp\left(-\frac{E_{a}}{kT}\right)$$

where σ is the ionic conductivity, σ_0_ is the pre-exponential factor, *E*_a_ is the activation energy, *k* is the Boltzmann constant, and *T* is the absolute temperature. In the Arrhenius plot of lnσ versus 1/*T*, the slope reflects *E*_a_, whereas the vertical intercept is governed by *σ*_0_. The pre-exponential factor σ_0_ depends on factors such as ionic mobility, carrier concentration, and microstructural continuity. This prefactor can be expressed as *σ*_0_=*μ*_0_ *nz*, where *μ*_0_ is the ionic mobility, *n* is the carrier concentration, and *z* is the charge state of the carrier.

In general, grain boundaries may affect both local carrier concentration and ionic mobility through space-charge formation and structural disorder.^12,46–48^ In the present samples, however, the YSZ composition is identical; therefore, the nominal oxygen-vacancy concentration is not expected to vary substantially among the samples. Thus, the increase in σ_0_ should be understood primarily as an increase in the effective transport prefactor, arising from reduced grain-boundary blocking and improved continuity of the out-of-plane conduction pathway, rather than from a substantial increase in carrier concentration.^49^

The potential influence of vertical columnar boundaries was further examined by ASTAR orientation mapping. As shown in Figure S10, neighboring YSZ columns in CZC–300+700 frequently exhibit subtle color gradients within similar color families, indicating similar crystallographic orientations and gradual local orientation variation rather than an abrupt, randomly oriented polycrystalline network. To quantify this local crystallographic variation, pixel-to-pixel orientation-deviation analysis was performed using the ASTAR maps (Figure S11). The violin plots show that CZC–300+700 exhibits a much narrower distribution centered near 0° than the single-layer YSZ films. The central 80% range of the signed local orientation deviation decreased from −10.6° to 11.7° for YSZ–300+700 and −3.6° to 4.5° for YSZ–700 to only −1.2° to 1.5° for CZC–300+700, demonstrating strongly suppressed local orientation fluctuations in the CZC structure.

To further compare locally significant orientation discontinuities, we quantified the scan-length-normalized density of adjacent-point orientation-deviation events exceeding 5°, a threshold selected within the low-angle regime based on the Read–Shockley description.^50^ This density decreased from 97.2 counts μm^–1^ for YSZ–300+700 and 48.1 counts μm^–1^ for YSZ–700 to 33.9 counts μm^–1^ for CZC–300+700, quantitatively supporting the reduced frequency of abrupt local orientation changes in the CZC structure. Since low-angle or more coherent grain boundaries have been reported to form narrower space-charge regions than highly incoherent high-angle boundaries, the reduced local disorientation in CZC–300 + 700 supports the interpretation that columnar boundaries exert limited detrimental effects on ionic transport. Therefore, the enhanced conductance is attributed primarily to reduced grain-boundary interruption along the out-of-plane transport direction, as quantified in Figure 4, with a possible secondary contribution from reduced columnar-boundary-related transport penalties.

## Conclusions

In this study, CZC trilayer electrolytes exhibited ionic conductance several times higher than that of single-layer YSZ, irrespective of the growth temperature. Notably, this enhancement was achieved in a system originally designed to improve chemical stability, highlighting an unexpectedly advantageous structure–property relationship.

Cross-sectional SEM and XRD inferred columnar growth and textured crystallographic orientation in the YSZ, GDC, and CZC films. TEM observations reveal a remarkable difference in the microstructure between the single-layer YSZ and the YSZ layer embedded within the CZC trilayer. Single-layer YSZ contains numerous fragmented columns and misoriented grain boundaries, including V-shaped column junctions that generated high-angle boundaries and clusters of fine grains, particularly near the substrate interface, regardless of the growth temperature. In contrast, the YSZ layer in the CZC trilayer consists of large, well-aligned columnar grains forming nearly uninterrupted crystallographic pathways along the out-of-plane direction. Even in CZC–300+700, the YSZ columns closely followed the orientation of the underlying GDC columns, indicating that epitaxial growth was maintained throughout the trilayer. ASTAR orientation mapping and FFT analyses confirm crystallographic alignment across all the layers, demonstrating coherent epitaxial growth and a substantially reduced grain boundary density, thereby minimizing the resistance from lattice discontinuities.

We found that two key factors enhanced the ionic conductance of the CZC layer, which was three to four times higher than the theoretically calculated value: (i) the suppression of intragrain boundary formation within each YSZ column in the CZC structure and (ii) reduced space charge effects due to predominantly low-angle, relatively coherent columnar boundaries. The negligible presence of intragrain boundaries in YSZ deposited on GDC helps preserve lattice continuity, thereby minimizing electrostatic and structural disruptions that impede oxygen-vacancy transport. The narrow column widths in CZC–300+700 (∼30 nm) could, in principle, promote overlapping space-charge regions, ASTAR orientation mapping indicates that most columnar boundaries in CZC exhibit low-angle misorientations, which suppress extended space-charge formation. In contrast, single-layer YSZ contains highly misoriented grain boundaries and fragmented grains that disrupt lattice continuity and impede ionic transport.

Overall, the enhanced ionic conductance of the CZC films arose from the combined effects of the decreased grain boundary density and improved structural coherence. The fluorite isomorphism and lattice similarity between YSZ and GDC facilitated column-by-column epitaxial growth, preserving crystalline continuity throughout the trilayer. This coherent microstructure effectively suppressed both the physical and electrostatic barriers to oxygen-ion transport. As a result, CZC thin-film electrolytes outperformed conventional single-layer YSZ while maintaining compatibility with low-temperature processing. These findings establish coherent multilayer design as an effective strategy for overcoming intrinsic transport limitations in thin-film electrolytes and provide clear guidance for the development of high-performance thin-film SOFCs.

## Experimental Methods

### Preparation of the Thin Films

Thin film samples were prepared by PLD. First, we separately compressed 8 mol % Y_2_O_3_-doped ZrO_2_ (YSZ) and 10 mol % Gd-doped CeO_2_ (GDC) powders at 200 MPa using a cold isostatic presser. The compressed powder pellets were sintered at 1400 °C for 5 h to obtain a dense PLD target. This target was ablated by a KrF excimer laser (λ = 248 nm, COMPEX Pro 201 F, Coherent) with an energy of 2.5 J·cm^–2^ at the target surface. To ensure film uniformity, we set the distance between the target and substrate to 5 cm. During deposition, the oxygen partial pressure was maintained at 6.67 Pa, and the substrate temperature was controlled at 300 °C (for GDC–300+700, YSZ–300+700, and CZC–300+700) or 700 °C (for GDC–700, YSZ–700, and CZC–700).

### Out-of-Plane Ionic Conductance Measurements

We determined the ionic conductance of the electrolytes deposited under different conditions through out-of-plane measurements using a two-probe configuration (inset, Figure 1) with a single-crystalline 0.7 wt % Nb-doped SrTiO_3_ (Nb:STO) (001) bottom electrode (10 × 10 mm^2^, 0.5 mm thickness) and a Pt (∼70 nm thickness) disk with a diameter of 1 mm as the top electrode. The bottom surface of the Nb:STO substrate was also coated by a Pt film (∼70 nm thick) to attain intimate contact with the Pt mesh placed on the heating block. Both the Pt disk and film were deposited by RF magnetron sputtering. Nb:STO has a high electronic conductivity (>143 Ω^–1^ cm^–1^ at room temperature), and it remains robust during high-temperature annealing. Notably, the differences in the thermal expansion coefficient between the STO (TEC_Nb:STO_ = 10.4 × 10^–6^ K^–1^) substrate and the GDC (TEC_GDC_ = 12.7 × 10^–6^ K^–1^) and YSZ (TEC_YSZ_ = 10.3 × 10^–6^ K^–1^) electrolytes were insignificant.^35,42^ The CZC trilayer was postannealed after all three layers were sequentially deposited without venting.

We placed the sample on the Pt mesh on top of the heating block in the microprobe station. The Pt microprobes contacted the top Pt electrode and the Pt mesh. We conducted EIS using a frequency response analyzer (FRA; SI1260, Solartron, UK) in a frequency range from 1 MHz to 0.1 Hz with a 100 mV perturbation amplitude under an air flow of 300 sccm. The ion conductance was derived from the fitted EIS arcs. The EIS spectra were fitted using an equivalent circuit consisting of a series resistor and a parallel RC element. The series resistance includes contributions from the measurement/contact configuration, as confirmed by control measurements using Pt/Nb/STO/Pt test structures.

### Characterization of the Thin Films

SEM (Inspect F, FEI, USA) was used to observe the cross-section of each thin film. The macroscopic crystal structures were evaluated by high-resolution XRD analysis (HR-XRD, DMAX2500, Rigaku, Japan) in *θ*–2*θ* mode using Cu Kα to determine the crystallographic relationship between substrate and film. We use a focused-ion beam (FIB; Ethos NX5000, Hitachi, Japan) to prepare the cross-sectional samples for TEM (Titan 300 kV, FEI, USA), which was employed to observe the crystal structure at the nanometer scale. We also recorded selected-area electron diffraction patterns during the HR-TEM analysis. Finally, we conducted ASTAR using TEM (Tecnai F20 200 kV, FEI, USA) with a 3 nm probe size and a precession angle of 0.7°.

## Supplementary Material



## Notes

Experimental details, supplementary structural characterization, AFM images, EIS spectra, Arrhenius analyses, TEM/ASTAR orientation maps, full-cell microstructural characterization, and supplementary tables (PDF)
