# Supplementary material for: Structural Origin of the Improved Ionic Conductance in CeO2–ZrO2–CeO2 Triple-Layer Electrolytes for Thin-Film Solid Oxide Fuel Cells
Source: ACS Nano. 2026 Jul 21;20(30):21081–92. doi: 10.1021/acsnano.6c02036 (PMC13450474; doi:10.1021/acsnano.6c02036)
Supplement: Supplementary file 1 [file nn6c02036_si_001.pdf]

Supplementary Information

Structural Origin of the Improved Ionic Conductance in CeO_2–ZrO_2–CeO_2 Triple-Layer Electrolytes for Thin-Film Solid Oxide Fuel Cells

Dongha Kim [‡,†], *Minyae Moon* [‡,§,†], *Hangyeol Jung* [‡,§], *Seongkook Oh* [‡,#], *Cheol-Hwee Shim* [▽], *Sungeun Yang* [‡,#], *Jong-Ho Lee* [‡,#], *Ji-Won Son* [‡,§,*], *Deok-Hwang Kwon* [‡,*]

[‡] Center for Energy Materials Research, Clean Energy Research Division, Korea Institute of Science and Technology (KIST), Seoul, Korea

[§] Department of Energy Environment Policy and Technology, Graduate School of Energy and Environment (KU-KIST Green School), Korea University, Seoul, Korea

[#] Division of Nano & Information Technology, KIST School, University of Science and Technology, Seoul, Korea

[▽] Advanced Analysis and Data Center, Korea Institute of Science and Technology (KIST), Seoul, Korea

†: These authors equally contributed.

[*] Corresponding Authors: Ji-Won Son/jwson@kist.re.kr; Deok-Hwang Kwon/dkwon@kist.re.kr

Temperature (°C)	GDC (Ω^{-1})	YSZ (Ω^{-1})	$G_c^{cal} = \frac{1}{R_c} = \frac{1}{R_{GDC,m} + R_{YSZ,m} + R_{GDC,m}}$ (Ω^{-1})	G_c^{Exp} (Ω^{-1})
200	1.34×10^{-3}	1.52×10^{-6}	4.73×10^{-6}	2.18×10^{-5}
225	3.18×10^{-3}	5.96×10^{-6}	1.85×10^{-5}	7.64×10^{-5}
250	6.91×10^{-3}	2.09×10^{-5}	6.48×10^{-5}	2.31×10^{-4}
275	1.41×10^{-2}	6.44×10^{-5}	1.99×10^{-4}	7.74×10^{-4}
300	2.67×10^{-2}	1.52×10^{-4}	4.69×10^{-4}	1.83×10^{-3}

Table S1. Conductances of 1.25-μm-thick GDC, YSZ, experimental CZC, and calculated CZC samples deposited at 700 °C, compared at various temperatures.

Temperature (°C)	GDC (Ω^{-1})	YSZ (Ω^{-1})	$G_c^{cal} = \frac{1}{R_c} = \frac{1}{R_{GDC,m} + R_{YSZ,m} + R_{GDC,m}}$ (Ω^{-1})	G_c^{Exp} (Ω^{-1})
200	9.65×10^{-4}	1.57×10^{-6}	4.89×10^{-6}	1.71×10^{-5}
225	2.49×10^{-3}	6.17×10^{-6}	1.92×10^{-5}	5.92×10^{-5}
250	5.87×10^{-3}	2.09×10^{-5}	6.47×10^{-5}	1.91×10^{-4}
275	1.28×10^{-2}	6.33×10^{-5}	1.96×10^{-4}	5.65×10^{-4}
300	2.61×10^{-2}	1.62×10^{-4}	4.99×10^{-4}	1.38×10^{-3}

Table S2. Conductances of 1.25-μm-thick GDC, YSZ, experimental CZC, and calculated CZC samples deposited at 300 °C and post annealed at 700 °C, compared at various temperatures.

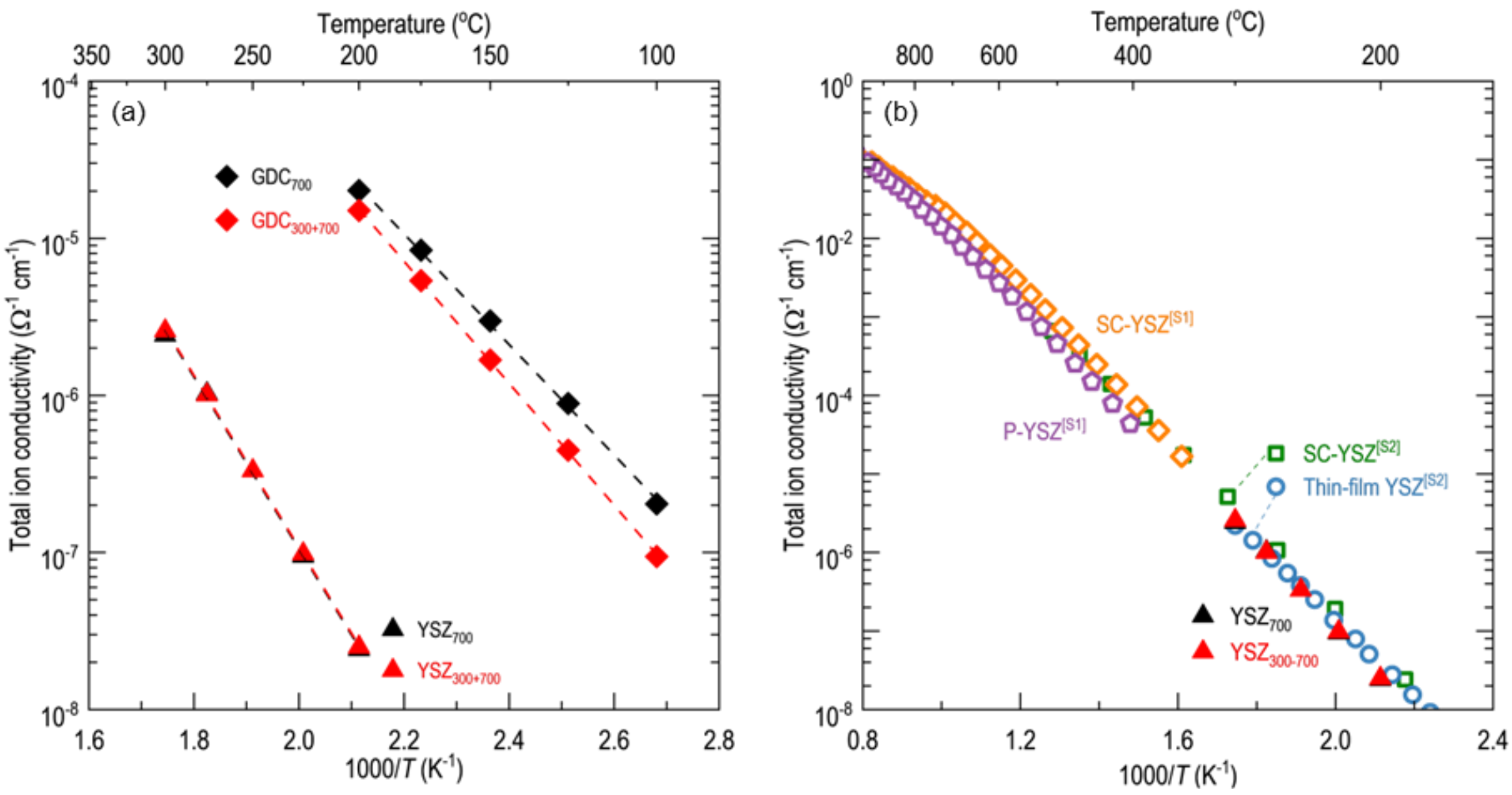

Figure S1. Arrhenius plots of the ionic conductivities of (a) single-layer YSZ and GDC samples and (b) comparison of the CZC-derived YSZ conductivity with reported YSZ values from the literature. SC, PC, and thin-film denote single-crystalline, polycrystalline, and thin-film YSZ, respectively.[S1, S2] The PC references correspond to sintered YSZ ceramics with grain sizes on the order of tens of micrometers.

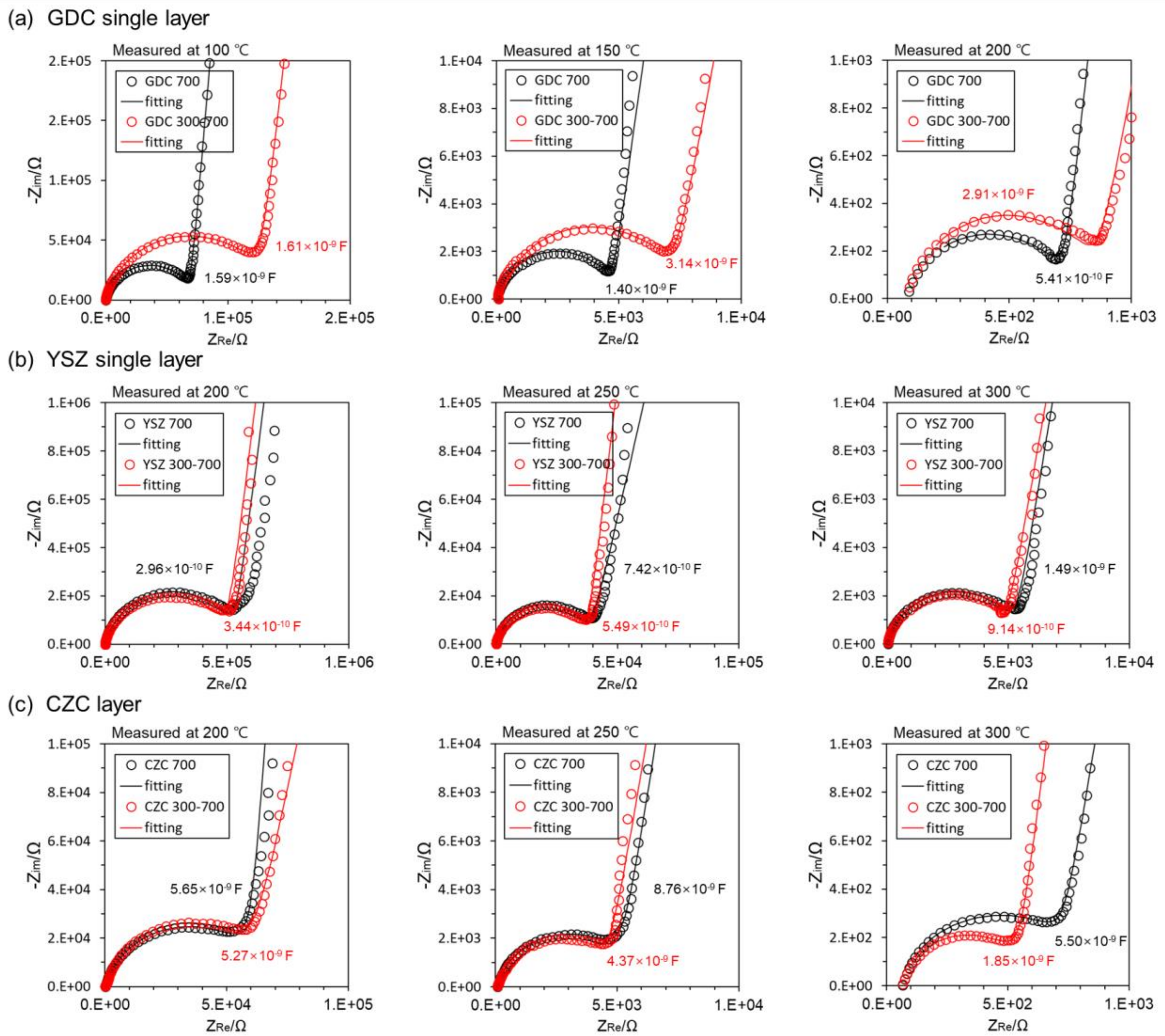

Figure S2. Electrochemical impedance spectra and fitted results of (a) GDC, (b) YSZ, and (C) CZC samples at various temperatures.

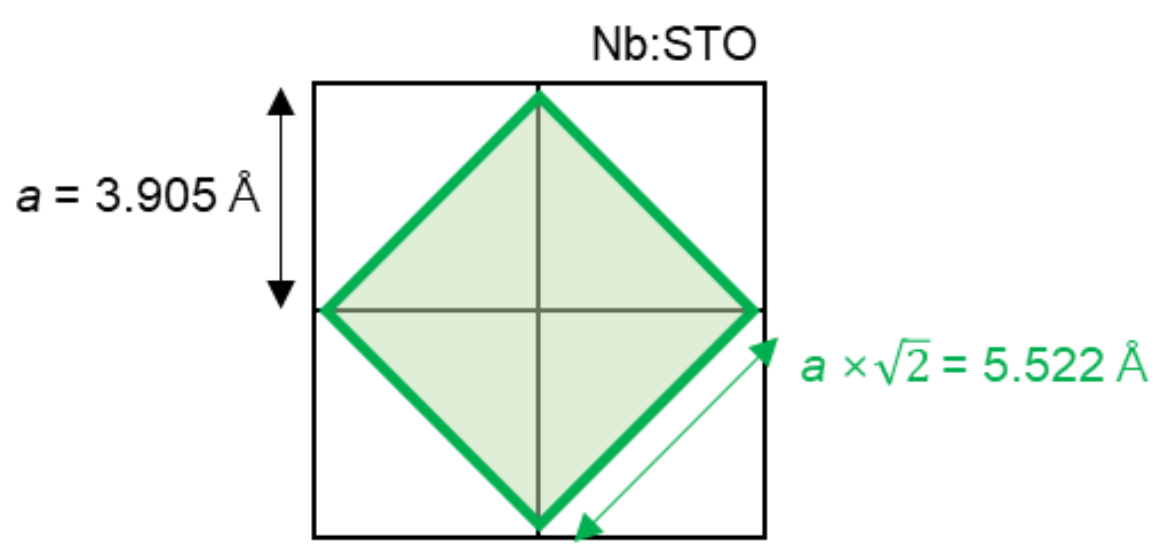

Figure S3. Schematics of the in-plane orientation of (001) Nb:STO and (001) GDC, illustrating the 45° rotated epitaxial relationship between Nb:STO and GDC.

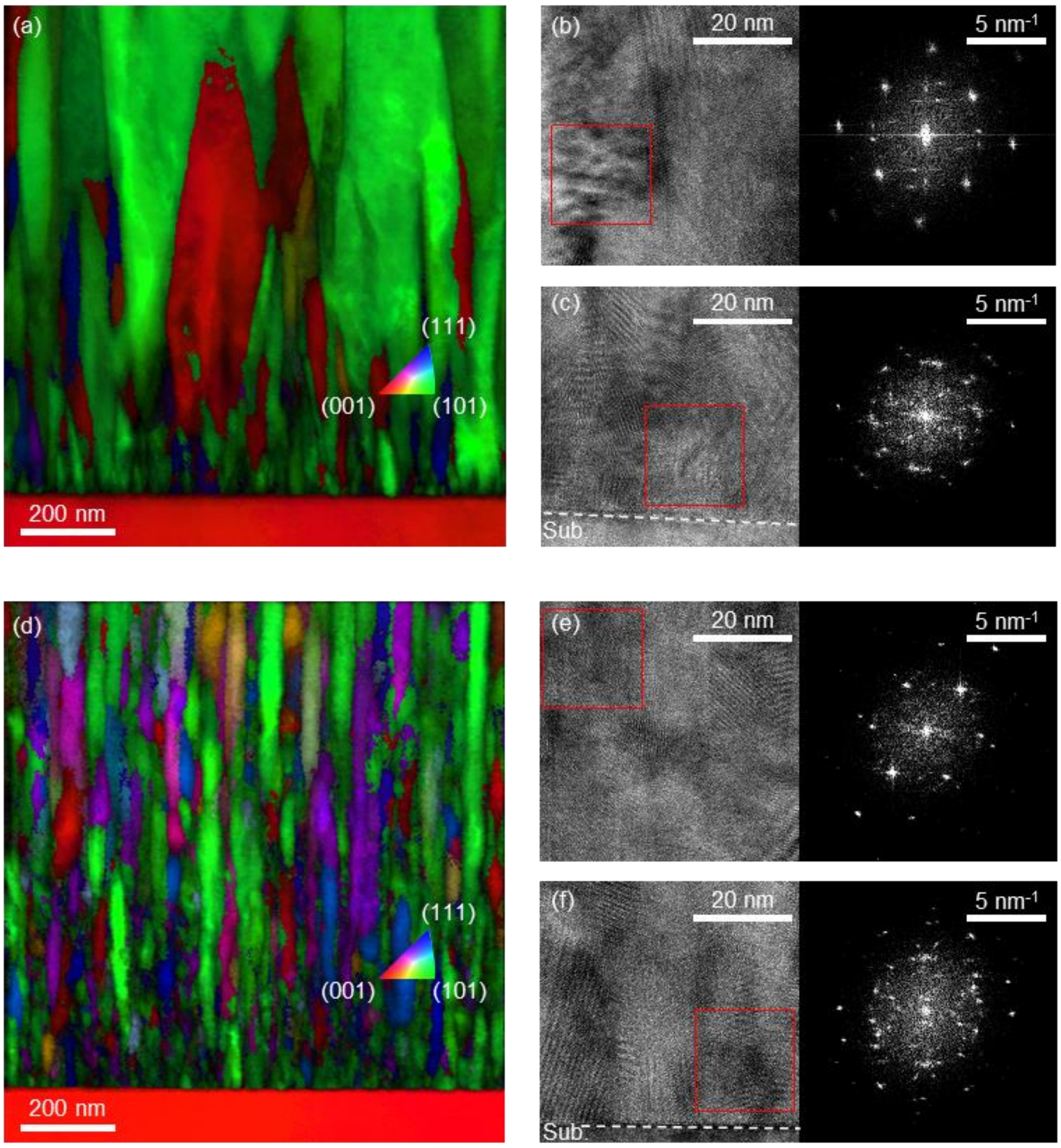

Figure S4. ASTAR orientation maps of (a)YSZ–700 and (b)YSZ–300+700, along with corresponding high-resolution TEM image and FFT pattern. The middle region (b, e) exhibit highly ordered grain alignment, whereas the areas near the substrate-film interfaces (c, f) display a more disordered structure.

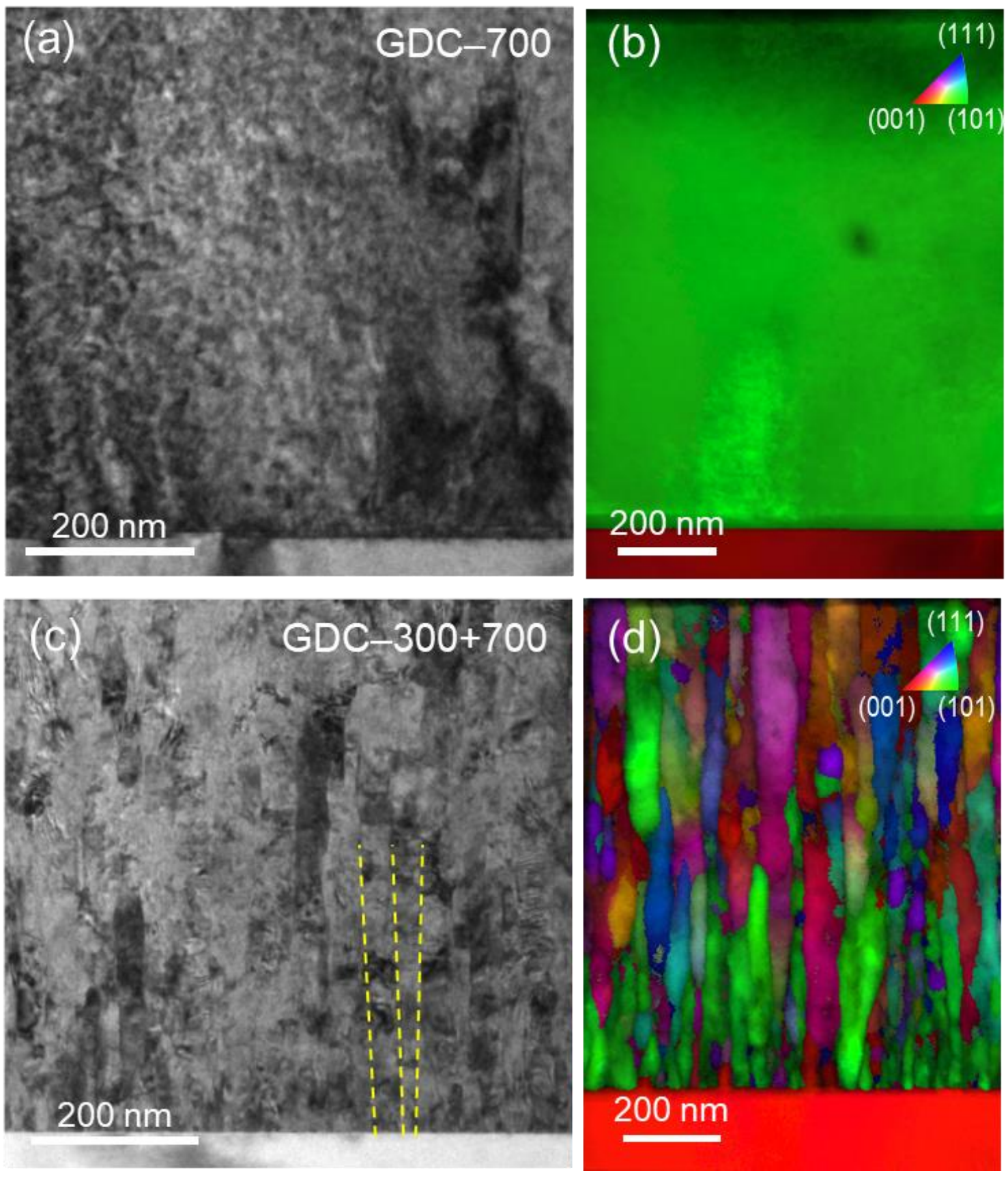

Figure S5. TEM images and crystal orientation maps of (a, b) GDC–700 and (c, d) GDC–300+700. The color code in (c) and (d) indicates the crystallographic grain orientation along the beam direction, aligned with the (001) orientation of Nb:STO.

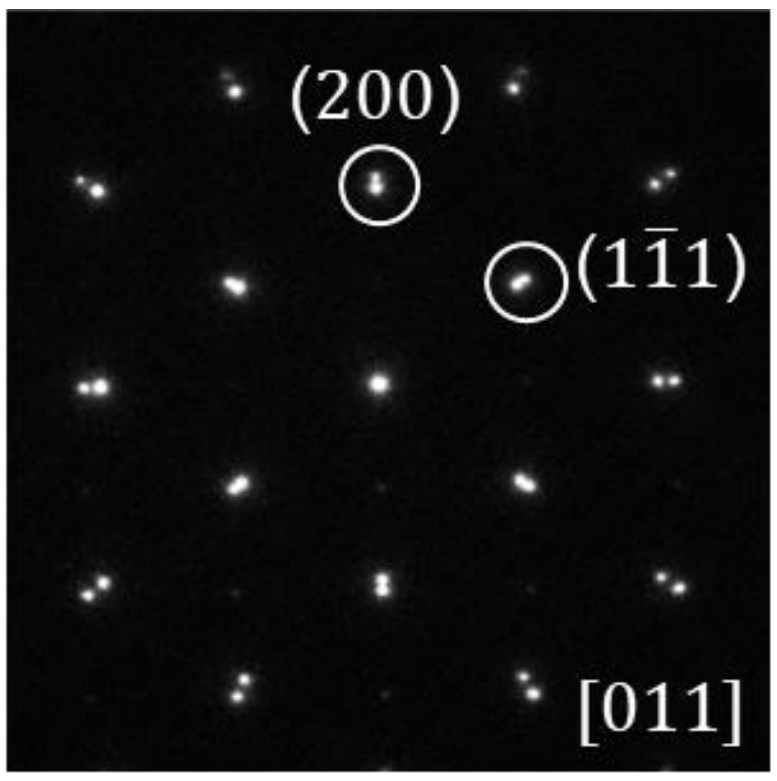

Figure S6 Selected-area electron diffraction patterns corresponding to the CZC–700 sample shown in Figure 2d.

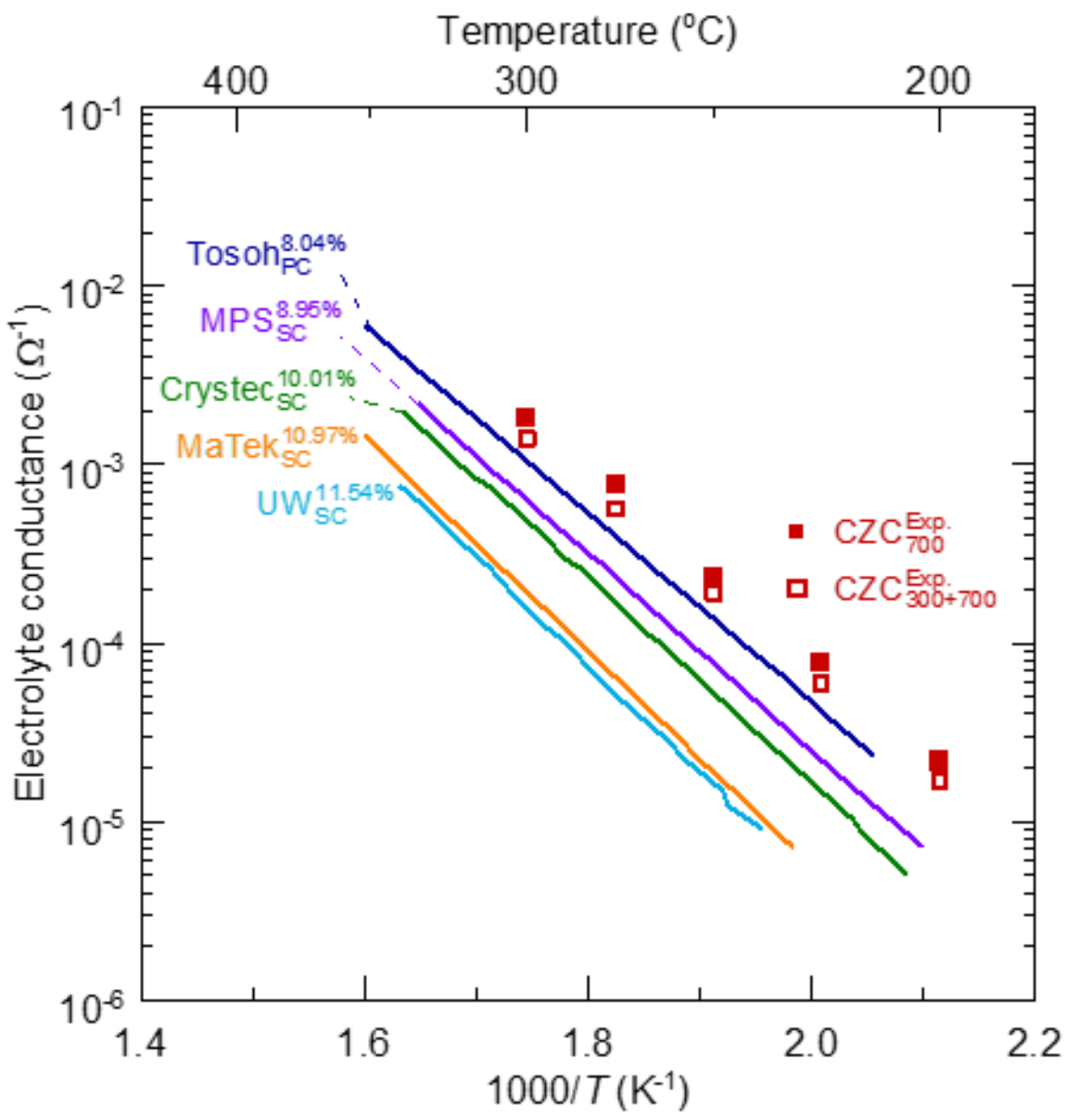

Figure S7. For a consistent comparison, all values are expressed in terms of conductance by converting reported conductivity values using a geometry of 400 nm thickness and a circular electrode with a diameter of 1 mm, consistent with the CZC device structure used in this study. Comparison of YSZ conductance values reported in the literature for different suppliers and compositions. Despite similar nominal compositions, the conductance of YSZ shows significant variation depending on the supplier, including Tosoh, MPI Solid State Research (MPS), Crystec, MaTek, and University Wafer (UW). The corresponding yttria concentrations are indicated for each dataset. The conductance values of CZC extracted in this work are overlaid for comparison. SC and PC denote single-crystalline and polycrystalline YSZ, respectively.[S3]

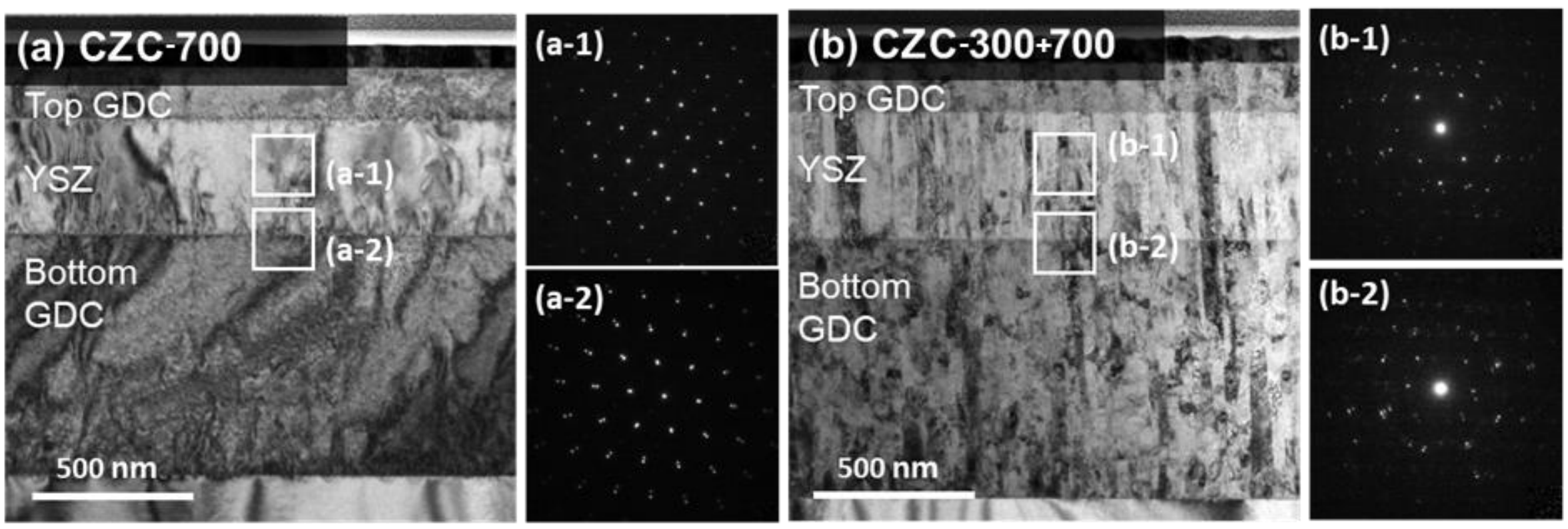

Figure S8. Selected-area electron diffraction patterns of (a) CZC–700 and (b) CZC–300+700. To investigate the effect of the bottom GDC layer, we also recorded images of the YSZ/GDC interface and the coarsened region. CZC–700 shows an epitaxial growth relationship between YSZ and the bottom GDC layer. Although CZC–300+700 is polycrystalline, the diffraction spots of GDC and YSZ show only a slight difference in the d-spacing while maintaining the same crystallographic orientation.

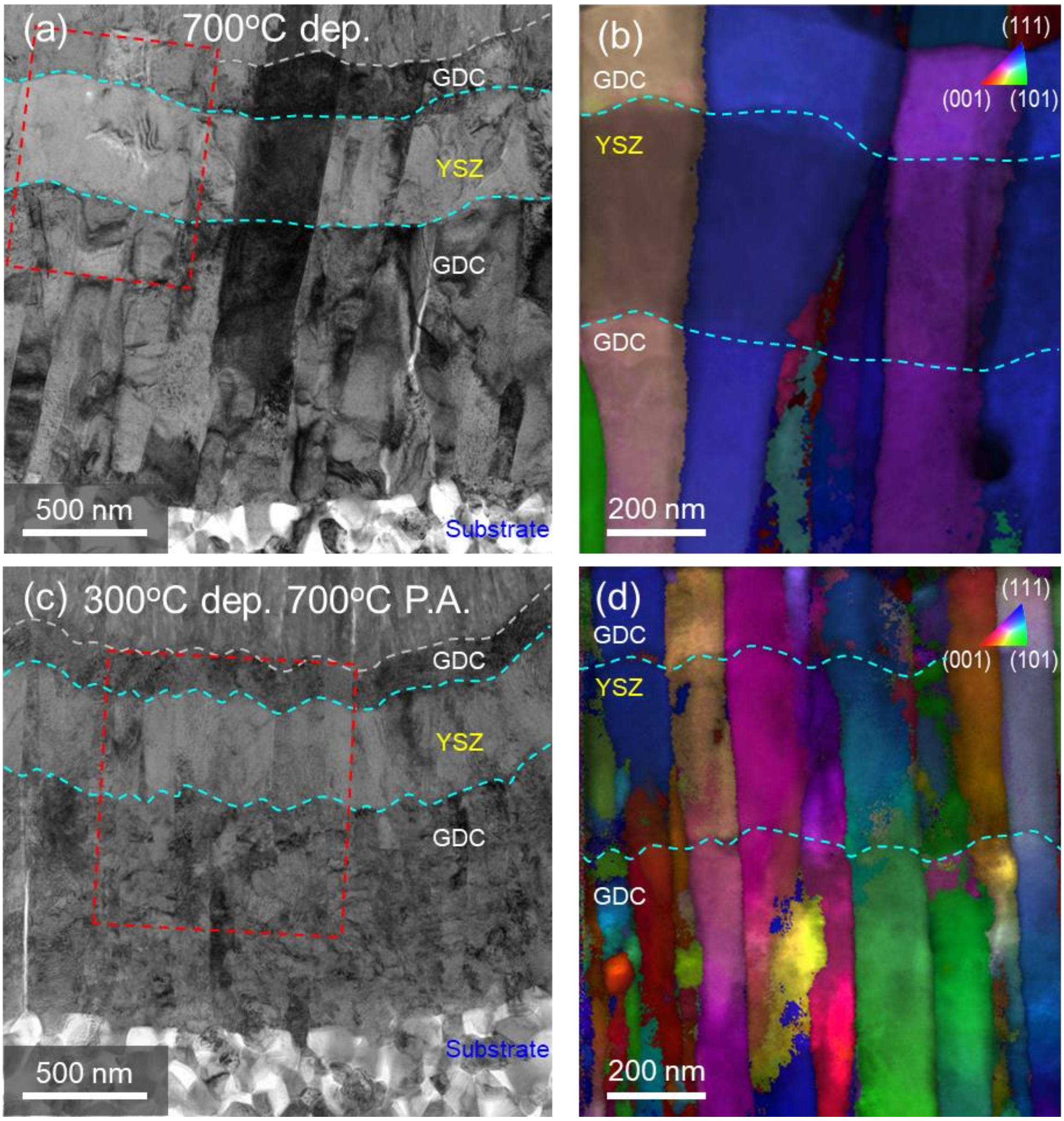

Figure S9. To evaluate the crystal structure of the electrolyte in a full cell, we constructed a CZC/nano-anode functional layer (40 vol% Ni-GDC)/anode functional layer/anode support structure based on 40 vol% Ni-YSZ. The CZC electrolyte deposition conditions were kept consistent with those used for the model-film samples, except for the underlying substrate/stack geometry. Within the full-cell samples, all processing conditions were identical except for the electrolyte deposition temperature. (a) TEM image and (b) crystallographic orientation map of the electrolyte layer in the reference full cell deposited at 700 °C. (c) TEM image and (d) crystallographic orientation map of the electrolyte layer in the full cell deposited at 300 °C and post-annealed at 700 °C. The ASTAR regions of interest (ROIs) are indicated by red dashed boxes in (a) and (c). The low-temperature sample exhibited narrower columnar grains than the reference cell. Nevertheless, owing to the templating effect of the bottom GDC layer, the YSZ interlayer maintained its epitaxial relationship and showed single-crystal-like columnar growth, which suppressed the formation of intragrain boundaries, consistent with the behavior observed in Fig. 3.

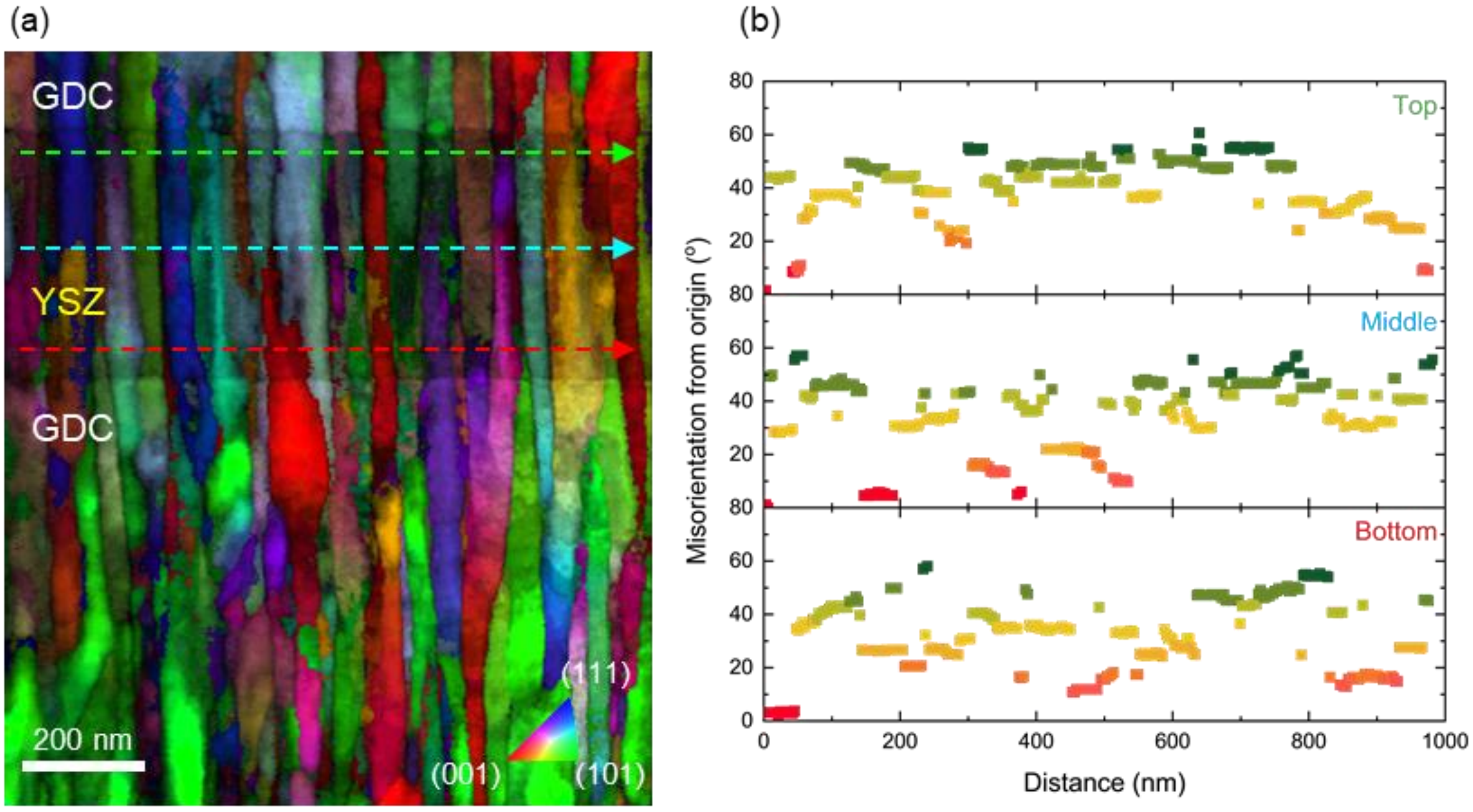

Figure S10. (a) ASTAR orientation map of the CZC–300+700 sample; inset: color key indicating the crystallographic orientation along the beam direction, referenced to the (001) orientation of Nb:STO. Intermediate colors represent intermediate crystallographic orientations between the principal directions shown in the legend. (b) Misorientation-from-origin line profiles of the YSZ, extracted along the dashed arrows in (a). The plotted values indicate angular deviation from the starting point of each line profile, rather than the direct misorientation angle of an individual grain boundary. These profiles highlight local orientation variation and local crystallographic correlation among neighboring columns. Note that the color coding of the points in the line profiles is unrelated to the ASTAR color scheme.

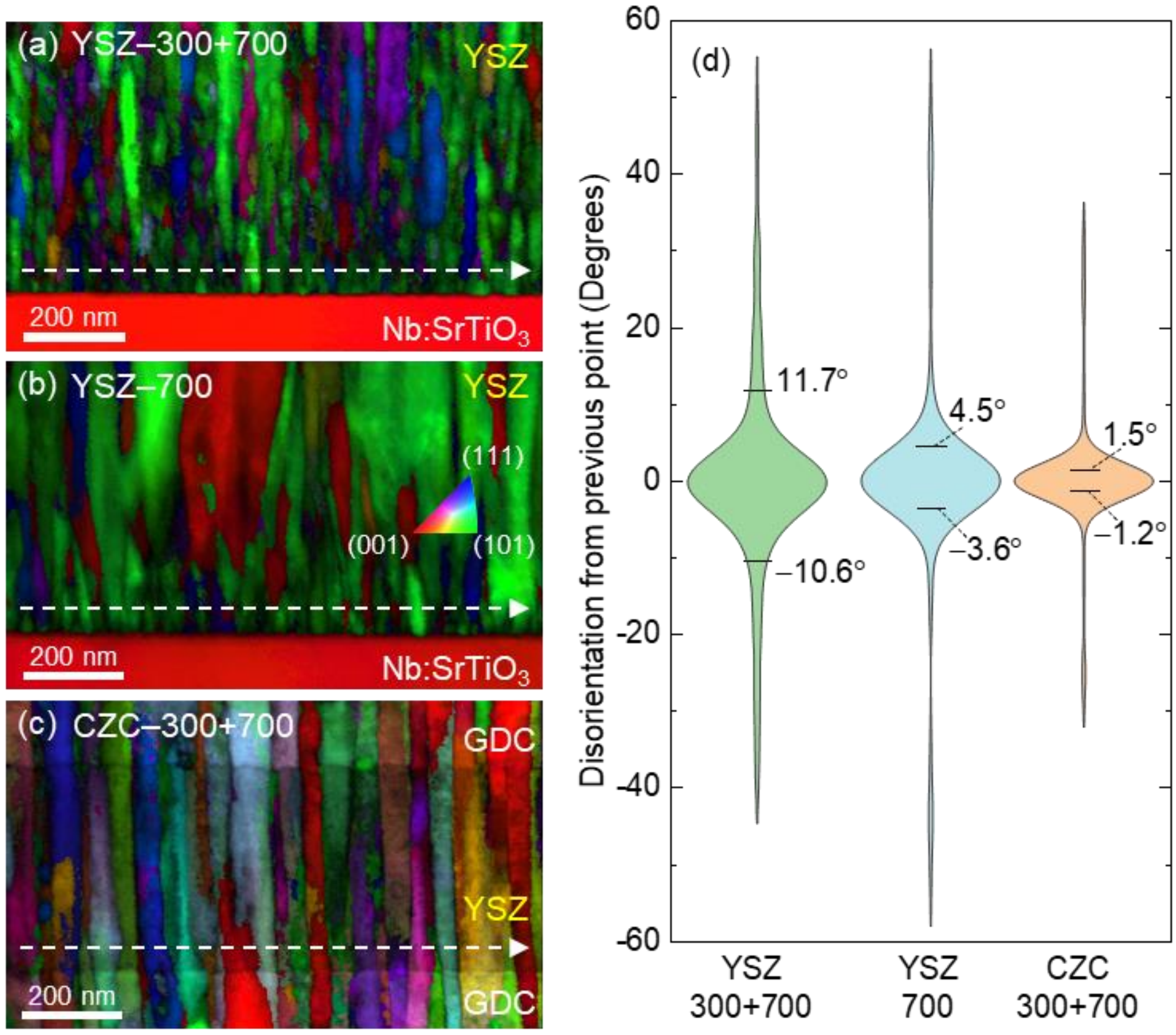

Figure S11. ASTAR orientation maps and corresponding local disorientation analysis for YSZ–300+700, YSZ–700, and CZC–300+700 near the interface region. (a–c) ASTAR orientation maps of (a) YSZ–300+700, (b) YSZ–700, and (c) CZC–300+700. The dashed arrows indicate the direction along which line profiles were extracted near the interface. (d) Violin plots of the local point-to-point disorientation angle distributions extracted along the dashed line profiles in (a-c). The distributions represent the angular difference between adjacent ASTAR measurement points along the scan direction and provide a semi-quantitative comparison of local crystallographic variation among the samples. The horizontal lines in each violin denote the 10th and 90th percentiles, corresponding to the central 80% of each dataset. CZC–300+700 exhibits a narrower distribution with fewer highly disoriented regions compared with the single-layer YSZ films, indicating more locally correlated columnar growth.

REFERENCES

S1. Badwal, S. P. S.; Electrical conductivity of single crystal and polycrystalline yttria-stabilized zirconia. *J. Mater. Sci.* **1984**, *19*, 1767-1776.

S2. Rivera, A.; Santamarıa, J.; Leon, C.; Electrical conductivity relaxation in thin-film yttria-stabilized zirconia. *Appl. Phys. Lett.* **2001**, *78*, 610-612.

S3. Ahamer, C.; Optiz, A. K.; Rupp, G. M.; Fleig, J.; Revisiting the temperature dependent ionic conductivity of yttria stabilized zirconia (YSZ). *J. Electrochem. Soc.* **2017**, *164*, F790-F803.
